# Supplementary material for: In situ investigation of the oxidation of a phospholipid monolayer by reactive oxygen species
Source: Biophys J. 2022 Oct 29;122(11):2007–22. doi: 10.1016/j.bpj.2022.10.040 (PMC10257017; doi:10.1016/j.bpj.2022.10.040)
Supplement: Document S1. Figs. S1–S3 and Table S1 [file mmc1.pdf]

Biophysical Journal, Volume *122*

Supplemental information

In situ investigation of the oxidation of a phospholipid monolayer by reactive oxygen species

Alexander P. Fellows, Mike T.L. Casford, and Paul B. Davies

Supplemental Information: *In situ* Investigation of the Oxidation of a Phospholipid Monolayer by Reactive Oxygen Species (ROS)

Alexander P. Fellows*,1 Mike T. L. Casford,1 and Paul B. Davies,1

1: Yusuf Hamied Department of Chemistry, University of Cambridge, Cambridge, CB21EW, United Kingdom

* apf36@cam.ac.uk

SFG Fitting Parameters

As mentioned in materials and methods, the SFG spectra were fitted according to the SFG equation with the terminal methyl groups having C_s symmetry. SFG simulations using this symmetry have shown that the in-plane mode contributes no intensity to the SSP polarisation combination and is negligible in PPP compared to the contributions from the out-of-plane mode.(1, 2) Therefore, as these are the only two polarisation combination employed in this work, for the purposes of fitting, only a single antisymmetric methyl resonance (r^-_{OP}) was fitted.

SFG spectra were fitted using an in-house MATLAB script using the parameters given in Table S1. The monolayers were assumed to adopt an all-trans geometry with 0° twist angles and have orientational distributions described by delta functions. By convention these assumptions are considered to be acceptable for well-packed monolayers and are commonplace in SFG investigations on lipids in the liquid-condensed phase.

Table S1: Parameters used for SFG fitting.

Parameter	Description	Value
n_1^{IR}	Refractive Index of IR in Air-Phase	1.00
n_1^{VIS}	Refractive Index of VIS in Air-Phase	1.00
n_1^{SFG}	Refractive Index of SFG in Air-Phase	1.00
n_2^{IR}	Refractive Index of IR in Sub-Phase	1.390 + 0.013i
n_2^{VIS}	Refractive Index of VIS in Sub-Phase	$1.326 + 1.3\times10^{-7}$i
n_2^{SFG}	Refractive Index of SFG in Sub-Phase	1.331

n_{layer}	Refractive Index of Monolayer	1.18
θ_{IR}	Incident IR Beam Angle	55°
θ_{VIS}	Incident VIS Beam Angle	60°
θ_{SFG}	Output SFG Beam Angle	59.27-59.31°
λ_{VIS}	VIS Beam Wavelength	532 nm
r	Single Bond Polarisability Derivative Ratio	0.0

SFG Analysis of the Phosphate Stretching Region

FTIR spectra shown in the manuscript led to the conclusion that the dominant chemical changes from ROS attack occur to the lipid head-group, in particular indicating changes to the phosphate and choline functional groups. It should, however, be noted that SFG spectra in both the SSP and PPP polarisation combinations showed no resonances from 1050-1600 cm^{-1}. (SFG spectra of phospholipids in this lower frequency range have been recorded previously(3) where bands such as the phosphate stretching modes have been observed.) The SFG spectrometer employed here, however, has a reduced output IR power at these lower IR frequencies (by ~1 order of magnitude compared to the C-H stretching region). Therefore, when combined with the relatively low acquisition rate (50 Hz), the usually weak phosphate bands become indistinguishable from the baseline.

Kinetic Mechanism of Fenton's Reagent

Catalytic activity in Fenton's reagent is often attributed to Fe^{2+} ions which can be regenerated from Fe^{3+} ions in steps 1 and 2 shown in the main text. The data in Figure 4 shows that the monolayer is sensitive to the sub-phase pH which causes precipitation of ferrous or ferric hydroxides. Furthermore, an increased concentration of $FeSO_4$ in the sub-phase (100 mg dm^{-3} cf. 10 mg dm^{-3}) showed a greater reduction in surface pressure from the same amount of injected H_2O_2 (e.g., by comparing Figures 3 and 5), pointing to non-catalytic behaviour.

Impact of Fe^{2+} Concentration on the Isotherm Changes

The effect of [$FeSO_4$] on the extent of lipid oxidation for equivalent amounts of injected H_2O_2 is shown in Figure S1 which plots isotherms recorded every 20 minutes after the injection of

H_2O_2 as presented in Figure 5 but for four further sub-phase $FeSO_4$ concentrations, ranging from 20-300 mg dm^{-3}.

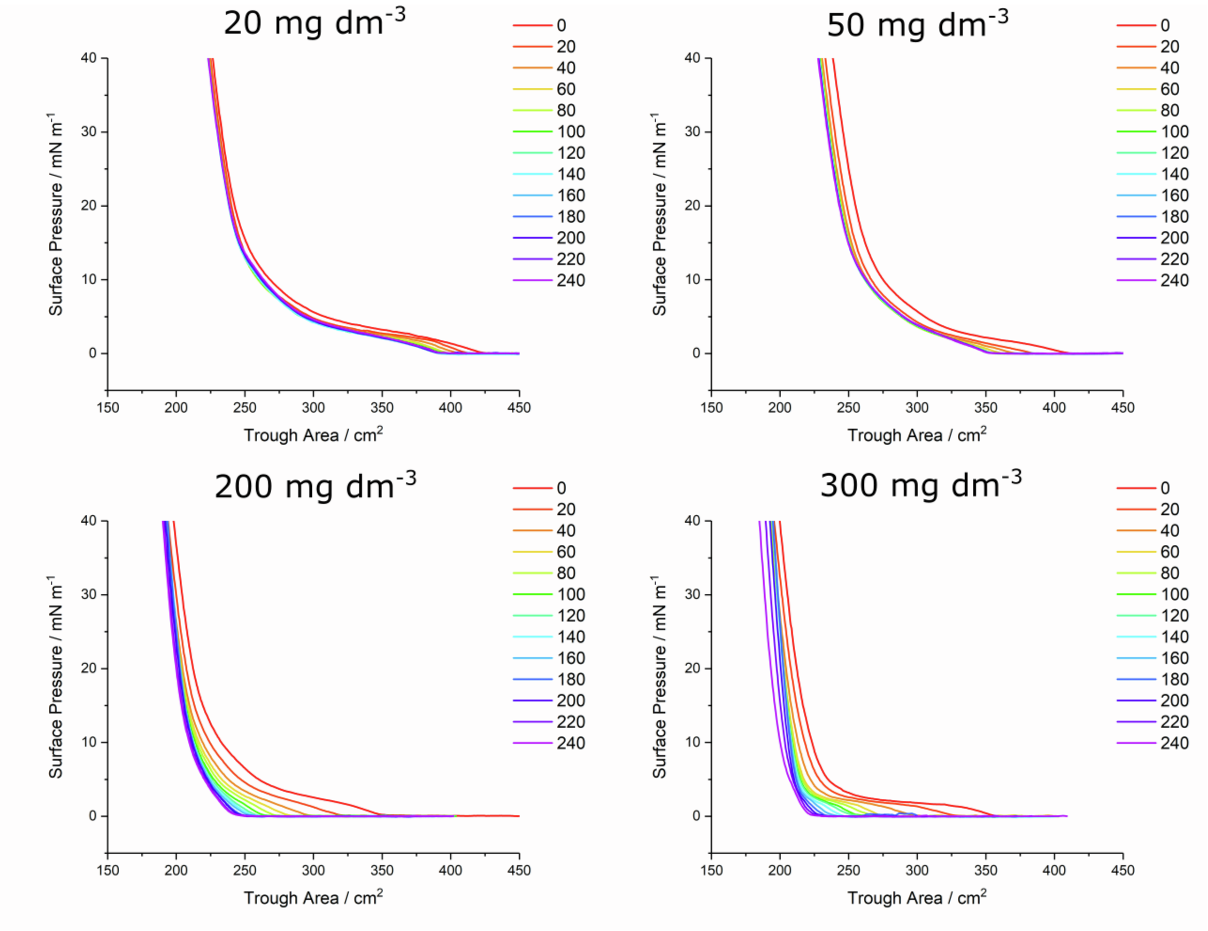

Figure S1: (Π – A) isotherms of a DPPC monolayer recorded at 20-minute intervals after the injection of H_2O_2 into the sub-phase containing different concentrations of $FeSO_4$, from 20 – 300 mg dm^{-3}.

Figure S2 shows the temporal change in trough area at 1 mN m^{-1} surface pressure (Figure S2a) and film compressibility (Figure S2b) for different $FeSO_4$ concentrations, the latter arising from the gradient between 30 and 40 mN m^{-1} from the data in Figure S1. Also shown are the equilibrium values reached for both parameters (Figure S2c and d), calculated from fits to an exponential decay of the plots in Figure S2a and taking the values reached after 4 hours in Figure S2b. The monolayer seems to become more condensed (occupying less surface area) and less compressible (showing a steeper gradient on compression). It is also worth noting that the change in compressibility is not monotonic, but the gradient appears to sharpen (increase) and then become shallower (decrease), as seen in Figure S2b, particularly at higher concentrations of $FeSO_4$. This correlates well with the multi-step kinetic mechanism

proposed from the temporal SFG and IRRAS analysis in the main text. Nevertheless, the differing concentrations of $FeSO_4$ seem to result in equivalent monolayer changes, only to different extents.

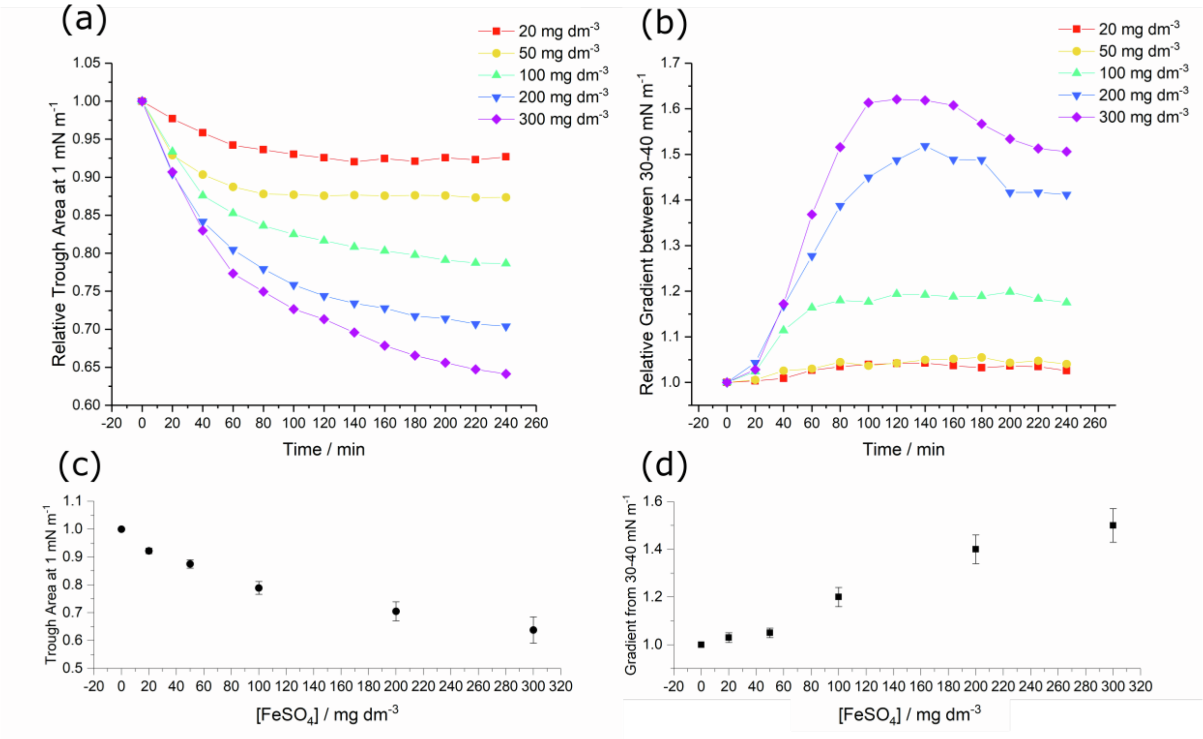

Figure S2: Analysis of the Π − A isotherms shown in Figure S1 for different concentrations of $FeSO_4$ in the sub-phase showing: (a) the change in relative trough area at 1 mN m^{-1}, (b) the relative change in isotherm gradient between 30-40 mN m^{-1}, (c) the equilibrium values of trough area at 1 mN m^{-1} calculated by fitting the plots in (a) to exponential decays, and (d) the final values of the gradient between 30 and 40 mN m^{-1}, taken as the values 4 hours after the injection of H_2O_2.

A Kinetic Description of Fenton Lipid Oxidation

Figure S2 shows that greater concentrations of aqueous ferrous ions in the sub-phase yield more substantial changes in the monolayer. Given that the ferrous salts are considered catalytic, it might be expected that a change in their concentration would result in a more rapid oxidation of the monolayer (i.e., higher decay constants), but the final changes should remain the same as they are dependent only on the amount of H_2O_2 available for conversion into ROS radicals. Evidently, this is not what is happening. Not only are the changes more significant for the same amount of H_2O_2 added, but the decay constants are smaller for higher $FeSO_4$ concentrations and take longer to reach their final values (Figure S2a). These observations could point to the

ferrous salts not acting solely as catalysts but are also being consumed in the reaction. This could be due to a combination of poor water solubility of ferric salts and hence precipitating out as ferric hydroxide, and not regenerating Fe^{2+} (i.e. $k_2 \rightarrow 0$), or a chain termination step as suggested in the Haber-Weiss reaction:

$$Fe^{2+} + OH + H^{+} \xrightarrow{k_3} Fe^{3+} + H_2O$$

where ferrous ions are converted to ferric ions and where OH radicals are destroyed rather than created, which would correspond to an inhibition step.

The precipitation of ferric hydroxide is supported by the observed colour change from nearly colourless to orange after the injection of H_2O_2. This implies that the conversion of Fe^{2+} to Fe^{3+} is irreversible. The decrease in observed decay constants at higher $FeSO_4$ concentrations (Figure S2a) indicates the significance of the inhibition step. By considering an elementary kinetic mechanism, based on the two steps given in the manuscript (with rate constants k_1 and k_2) and third step presented above (with rate constant k_3), as well as the direct lipid oxidation step:

$$L + OH \xrightarrow{k_L} L_{ox}$$

it is straightforward to show that the rate of loss of lipid is given by Eq. S1. In the absence of the inhibition step (i.e., $k_3 = 0$), the rate is simply determined by step 1 and is dependent only on the concentrations of Fe^{2+} and H_2O_2. However, including the inhibition step reduces the reaction rate, where it is clear that higher Fe^{2+} concentrations inhibit the oxidation of lipids (as $[Fe^{2+}]$ is included in the k_3 term in the denominator). Under conditions where ferric hydroxide is precipitated, the reaction becomes dependent on the *initial* concentration of $FeSO_4$ since ferrous ions cannot be regenerated.

$$\frac{d[L]}{dt} = -\frac{k_1 k_L [L][Fe^{2+}][H_2O_2]}{k_3[Fe^{2+}][H^{+}] + k_L[L]} \qquad (S1)$$

To further demonstrate how the extent of oxidation depends on both the $FeSO_4$ and H_2O_2 concentrations, the resulting pressure drop of a DPPC monolayer starting at 20 mN m^{-1} was monitored whilst varying the concentrations of each reactant in the sub-phase (i.e., both

the starting $FeSO_4$ concentration and the amount of H_2O_2 added). The resulting behaviour of the film is shown in Figure S3. On increasing the initial $FeSO_4$ concentration, the increase in the pressure drop becomes smaller, in agreement with the above analysis which suggested that Fe^{2+} cannot be regenerated and therefore inhibits lipid oxidation. Significantly, Figure S3b shows there is a levelling off of saturation in the pressure drop at high H_2O_2 concentrations, suggesting that H_2O_2 is in excess when added in these amounts and that the Fe^{2+} in the reaction mixture has been consumed.

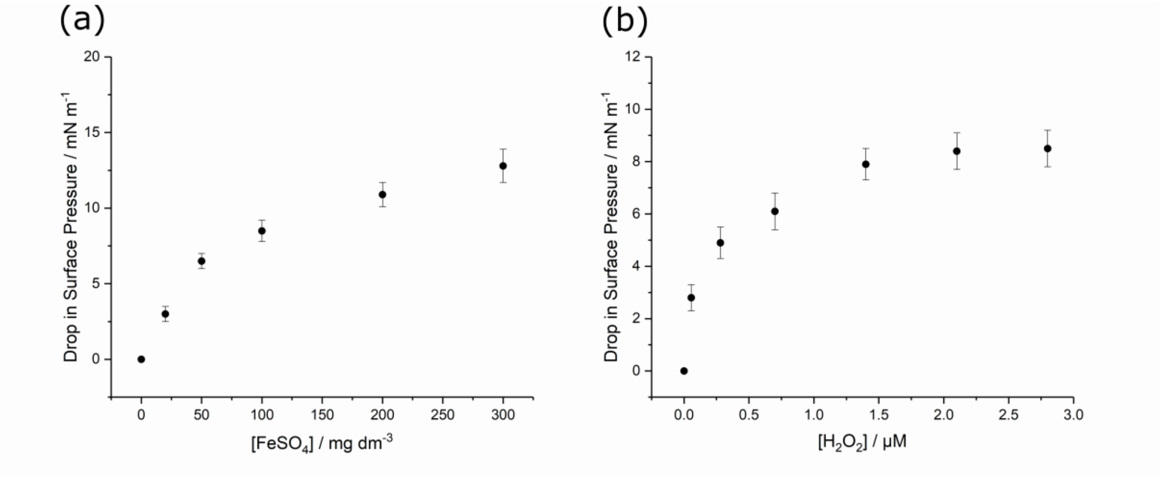

Figure S3: Drop in surface pressure for a 20 mN m^{-1} DPPC monolayer at the air- $FeSO_{4(aq)}$ interface in response to the injection of H_2O_2 into the sub-phase showing: (a) the effect of changing the sub-phase $FeSO_4$ concentration, and (b) the effect of changing the amount of H_2O_2 injected (converted into the equivalent concentration in the sub-phase).

References

1. Feng, R.J., X. Li, Z. Zhang, Z. Lu, and Y. Guo. 2016. Spectral assignment and orientational analysis in a vibrational sum frequency generation study of DPPC monolayers at the air/water interface. *J. Chem. Phys.* 145:244707.

2. Fellows, A.P., M.T.L. Casford, and P.B. Davies. 2021. Orientation Analysis of Sum-Frequency Generation Spectra of Di-chain Phospholipids: Effect of the Second Acyl Chain. *AIP Adv.* 11:045119.

3. Ma, G., and H.C. Allen. 2006. DPPC Langmuir monolayer at the air-water interface: Probing the tail and head groups by vibrational sum frequency generation spectroscopy. *Langmuir*. 22:5341–5349.
