# Supplementary material for: In situ investigation of the oxidation of a phospholipid monolayer by reactive oxygen species
Source: Biophys J. 2022 Oct 29;122(11):2007–22. doi: 10.1016/j.bpj.2022.10.040 (PMC10257017; doi:10.1016/j.bpj.2022.10.040)
Supplement: Document S2. Article plus supporting material [file mmc2.pdf]

Biophysical Journal
Article

Biophysical Society

In situ investigation of the oxidation of a phospholipid monolayer by reactive oxygen species

Alexander P. Fellows,[1,*] Mike T. L. Casford,[1] and Paul B. Davies[1]
[1]Yusuf Hamied Department of Chemistry, University of Cambridge, Cambridge, UK

ABSTRACT The oxidation of membrane lipids has been widely studied for several decades owing to its significance in biological systems. However, despite its damaging physiological impact and its known role in many diseases, relatively little is understood about the specific structural consequences of oxidative action, particularly in vivo. In this work, a combination of sum-frequency generation spectroscopy, surface tensiometry, and surface-selective infrared spectroscopies are used to gain deeper insight into the oxidation of phospholipids by reactive oxygen species generated in situ. Oxidation is achieved by employing the Fenton reaction to convert physiological levels of H_2O_2 into OH and HO_2 radicals in proximity to the headgroups of lipid monolayers at the air-water interface. By temporally monitoring the surface tension and spectroscopic changes at the interface as the oxidation proceeds, the impact of oxidation on the structure, conformation, and intermolecular interactions within the membrane has been revealed.

SIGNIFICANCE Given the ubiquitous presence of oxidation in biological systems, particularly in cell membranes where it has malign consequences, understanding its influence on the physical and chemical properties of the membrane is critically important. The work presented here uses a combination of surface tensiometry, sum-frequency generation spectroscopy, attenuated total reflection spectroscopy, and infrared reflection absorption spectroscopy to elucidate the oxidation mechanism occurring in situ in a lipid monolayer used to mimic a cell leaflet. It gives significant and original insights into specific chemical, structural, and conformational changes occurring in a lipid membrane exposed to the reactive oxygen species commonly found in physiology. Consequently, this work leads to deeper understanding of the role of oxidation in a variety of biological contexts.

INTRODUCTION

Lipids play a critical role in a multitude of physiological processes and particularly in cell membranes (1). It follows that any change in the lipid structure due to oxidation can alter the behavior of the membrane itself, eventually leading to premature apoptosis (2). Hence, the oxidation of lipids has been a significant topic of interest for many years (3–9). The oxidation of lipids in membranes is caused by exposure of their headgroups in the bilayer to reactive oxygen species (ROS). Studying how ROS attack or penetrate lipid headgroups is key to understanding the role of ROS in the oxidative stress of cells.

ROS are short-lived in aqueous environments, so in order to investigate oxidation pathways experimentally they must be generated and examined in situ (10). To achieve this several possibilities exist, namely using ionizing radiation either to split water into OH radicals (also producing other ROS species simultaneously) (11) or photochemically, producing ROS using photosensitizers (12) such as methylene blue (13–15) or ketones in the presence of alcohols (16). However, the exposure of lipids to intense ionizing radiation has the potential to perturb the lipid layers themselves. For this reason, it is important to avoid introducing intense external stimuli such as UV light to generate ROS. Other methods for ROS generation that have been used previously exploit heavy metals (17) or nanoparticles (18–20) in solution, but these systems are generally well removed from physiological relevance.

One particular process for generating ROS that is much more physiologically significant is the Fenton reaction, which transforms H_2O_2 into OH and HO_2 radicals using aqueous iron ions as catalysts (21–28). Fenton's reagent itself is a mixture of H_2O_2 and aqueous $FeSO_4$, and has been used previously to investigate oxidation by ROS due to its

Submitted August 30, 2022, and accepted for publication October 26, 2022.
*Correspondence: apf36@cam.ac.uk
Editor: Ilya Levental.
https://doi.org/10.1016/j.bpj.2022.10.040
© 2022 Biophysical Society.
This is an open access article under the CC BY license (http://creativecommons.org/licenses/by/4.0/).

biological relevance (29–33). The associated reactions are as follows:

$$Fe^{2+} + H_2O_2 \xrightarrow{k_1} Fe^{3+} + OH + HO^- ,$$

$$Fe^{3+} + H_2O_2 \xrightarrow{k_2} Fe^{2+} + HO_2 + H^+.$$

The Fenton reaction represents a mechanism for producing species naturally present in vivo. since both ferrous and ferric ions are found in biological systems (25–28,34). Sickle cells, for example, are thought to be exposed to greater oxidative stress due to the autoxidation of Fe^{2+} in the mutant hemoglobin (35,36).

In this work, Fenton chemistry is used to generate ROS beneath a phospholipid monolayer at the air-water interface, mimicking the exposure of the headgroups of lipids in a cell membrane leaflet. The oxidation mechanism is then probed temporally during the oxidative exposure by surface tensiometry, sum-frequency generation (SFG) spectroscopy, attenuated total internal reflection (ATR) spectroscopy, and infrared reflection absorption spectroscopy (IRRAS). Because of its surface specificity and sub-monolayer sensitivity, SFG has been highly useful in characterizing the chemical, structural, and conformational changes that occur to monolayers at the air-water interface when they undergo oxidation (37). Specifically, SFG, along with surface pressure measurements, has been used to study the oxidation of pulmonary lipids on exposure to ambient ozone, where any unsaturation in their structure leads to their rapid instability at the air-water interface on exposure to levels of ozone of just ≈10 ppb (38–45). SFG analysis showed clear structural changes in the monolayers in the form of gauche defects and disruption to the molecular packing. Furthermore, it was also possible to elucidate the mechanism of oxidation and chemical changes occurring at the interface (43) by comparison with the SFG spectra of proposed oxidation products, the SFG conclusions subsequently being confirmed using liquid chromatography-mass spectrometry (LC-MS) (45).

MATERIALS AND METHODS

Lipids

1,2-Dipalmitoyl-*sn*-glycero-3-phosphocholine (DPPC), d_{75}-DPPC (>99% deuteration), and 1-palmitoyl-2-oleoyl-glycero-3-phosphocholine (POPC) were obtained from Avanti Polar Lipids (Birmingham, AL) and cholesterol from Sigma-Aldrich (St. Louis, MO), all with >99% purity, and used as received. The structures of DPPC and POPC are given in Fig. 1.

Formation of monolayers

The lipid monolayers were formed at the air-water (or N_2-water) interface by depositing ≈20 to 40 μL aliquots of 1 mg mL^{-1} chloroform solutions onto the surface of a Langmuir-Blodgett (LB) polytetrafluoroethylene

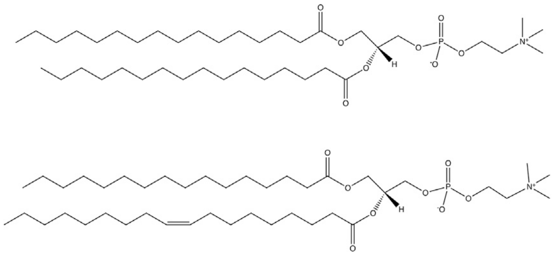

FIGURE 1 Structure of DPPC and POPC lipids.

(PTFE) trough filled with Millipore water (25°C ± 1°C, 18.2 MΩ cm, < 3 ppb total organic carbon). The trough was cleaned by wiping with both water- and chloroform-soaked tissue and repeatedly aspirating the interface until the surface pressure was <0.1 mN m^{-1} at full compression. Once clean, the lipid was deposited and left for at least 30 min to allow the solvent to evaporate. When using POPC, the oxidation of the monolayer due to low-level ozone (mentioned above) was avoided by enclosing the LB trough in a Perspex and high-density foam box constructed in-house and purging with humidified N_2 (dry N_2 bubbled through a sintered Dreschel bottle filled with Millipore water). The nitrogen purge was switched on after aspirating (cleaning) the interface and left for 30 min before deposition of the monolayer. Ozone levels were measured using an Aeroqual Series 200 ozone monitor (Aeroqual, Auckland, New Zealand) fitted with a 0–0.150 ppm O_3 sensor head.

For ATR measurements, the monolayers were LB deposited directly onto the ATR crystals by immersing the crystals prior to deposition of the monolayer, compressing the film, and retracting the crystal through the interface while maintaining constant surface pressure via a feedback loop. Casting the monolayer onto a solid substrate has the potential to alter its structure, and thus considerable caution was exercised when forming LB films. Here, the crystal was slowly withdrawn through the interface at a speed of 5 mm min^{-1} to minimize the disruption to the monolayer, and the transfer ratios were found to be unity within one standard deviation. Therefore, by following this protocol, it is safe to be confident that any structural perturbations are small.

Generating ROS

ROS were created in situ using Fenton chemistry by injecting 100 μL (unless otherwise specified) of H_2O_2 into the sub-phase which contained aqueous $FeSO_4$ (ranging from 10 to 300 mg dm^{-3}). For pH-dependence measurements, the pH of the sub-phase was adjusted using either H_2SO_4 or NaOH.

SFG spectroscopy

SFG spectra were recorded on a picosecond narrow-band spectrometer (Ekspla, Vilnius, Lithuania), which generates 29-ps 532-nm pulses at 50 Hz from the second harmonic of a mode-locked Nd:YAG laser. The visible beam (incident at 60° to the surface normal) was overlapped spatially and temporally in a co-propagating geometry with a tunable infrared beam in the range 1050–4000 cm^{-1} (incident at 55° to the surface normal) produced by an optical parametric oscillator (OPO).

SFG spectra were recorded in the SSP or PPP polarization combinations in either the frequency or temporal domains. Spectra were obtained with 2 cm^{-1} frequency resolution at either 200 or 1000 acquisitions per point (4 or 20 second acquisitions), depending on the desired temporal resolution (time required to record each spectrum). Temporal analysis was performed with 2000 acquisitions per point (40 second resolution) to achieve high signal/noise ratios.

Spectra were fitted using the SFG equation (46,47) based on C_s methyl symmetry and Hirose's bond additivity model (48–50). The full set of fitting parameters and a greater discussion of the fitting is given in supporting material. Ultimately, the loss of degeneracy of the two antisymmetric methyl stretching modes results in separate in-plane (r^-_{IP}) and out-of-plane (r^-_{OP}) methyl resonances, with the latter being the only one of significance in either SSP or PPP polarization combinations. From here on, therefore, no mention will be made of the in-plane mode in SFG discussions. Additionally, the effect of two different methyl groups in the lipid (in principle with different orientational distributions) was ignored, due to any spectral differences arising from the symmetric-tail, well-packed monolayers being below the noise threshold in this work (51).

Finally, the influence of the incident IR intensity on the monolayer structure (due to laser-induced heating) was assessed prior to undertaking measurements. No intensity variations were observed, as expected for monolayers with the high packing densities used here (52).

Surface tensiometry

Surface pressure measurements were recorded on a custom-made Langmuir trough that was fitted with a type PS4 surface pressure sensor (NIMA, Nottingham, UK) and designed for simultaneous SFG measurements. A motor-driven PTFE barrier was used to compress the monolayer to the desired surface pressure at 10 cm^2 min^{-1}. Once compressed, the monolayers were left for $\approx$30 min to equilibrate before performing experiments.

FTIR spectroscopy

Fourier transform infrared (FTIR) spectra of the monolayers were recorded in either ATR or IRRAS experimental geometries on a Bruker Vertex V70 instrument equipped with a liquid nitrogen-cooled mercury cadmium telluride detector (Bruker, Billerica, MA). The spectrometer was purged with dry, CO_2-scrubbed air to minimize any atmospheric absorption. Each spectrum was recorded with 4 cm^{-1} resolution with 1000 co-averages. During measurement, the rate of purge flow was controlled to minimize atmospheric contributions (matching the environment used for the background recording).

ATR measurements were performed in a multi-reflection geometry (25 reflections) using 52 $\times$ 20 $\times$ 2 mm 45° germanium trapezoidal prisms. The prisms were cleaned using soap, rinsed thoroughly with water, dried under dry nitrogen, and exposed to UV-ozone cleaning for 30 min. After recording a background spectrum, the prism was dipped into the Langmuir trough for LB-deposition of the monolayer.

IRRAS was performed directly off the air-water interface using a custom-made Langmuir trough and a variable angle reflection accessory (Specac, Orpington, UK), with the angle set to 50° to optimize the absorbance intensity (53).

RESULTS AND DISCUSSION

Effect of H_2O_2 on lipid temporal stability

As mentioned above, unlike saturated lipids which show long-term stability, unsaturated lipids are unstable in air due to ozonolysis by ambient ozone. This is demonstrated for POPC in Fig. 2 *a*, which shows the temporal variation in the surface pressure of a 20 mN m^{-1} film when a N_2 atmosphere initially covering it is interrupted by ambient air containing $\approx$30 $\pm$ 10 ppb of ozone. Once exposed to O_3, the surface pressure rises due to oxidation of the film.

H_2O_2 is an example of an ROS that can be formed in vivo and has been suggested to play a key role in lipid oxidation (54, 55). However, as seen in Fig. 2 *b*, a POPC monolayer under N_2 shows no change in surface pressure on adding 100 μL of H_2O_2 into the sub-phase. This is equivalent to a

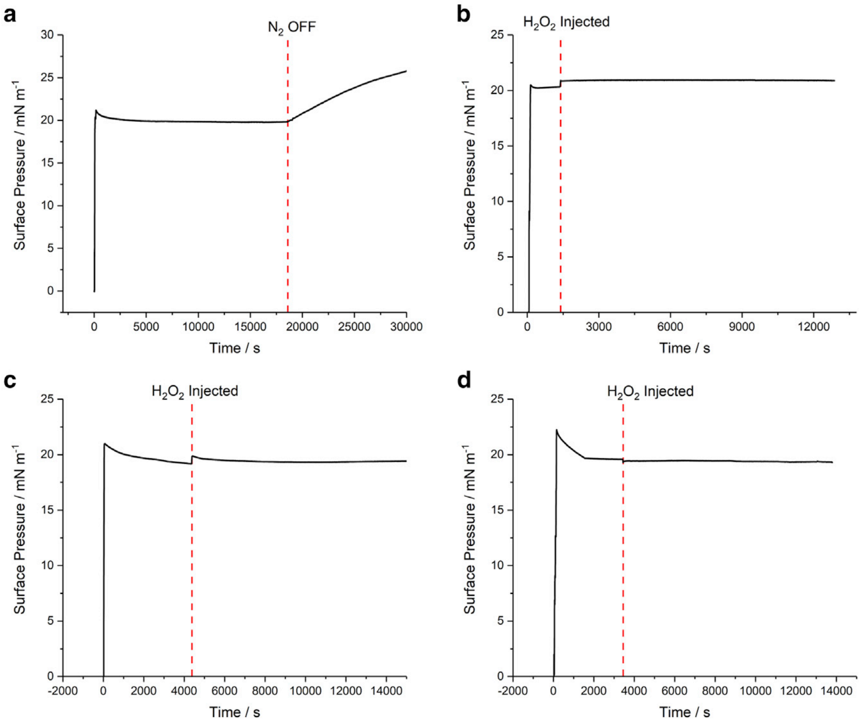

FIGURE 2 Temporal surface pressure analysis of lipid monolayers initially at 20 mN m^{-1} at the N_2-water interface, showing: (*a*) a POPC monolayer with the N_2 purge switched off, and monolayers of (*b*) POPC, (*c*) 1:1 POPC and DPPC, and (*d*) 1:1:1 POPC, DPPC, and cholesterol, each with 100 μL of H_2O_2 injected into the sub-phase. To see this figure in color, go online.

concentration of ≈ 2.8 μM, which is within the range typically found in blood plasma and thus of physiological relevance (56). Consequently, Fig. 2 *b* suggests that, although H_2O_2 may play a significant role in lipid oxidation, it is insufficient on its own to disrupt the membrane. This is supported by studies that examined the thermodynamics of different ROS entering a lipid bilayer, where it was found that H_2O_2 possessed the highest energy barrier to penetrating the headgroup (57).

It is well known that real membranes are much more complex than single-component monolayers formed in the laboratory which, hence, represent a significant simplification. Instead, multi-component lipid monolayers (and bilayers) are often used. These can form lipid rafts (domains), which play an important role in membrane structure and function (58). Lipid mixtures known to form such domains include combinations of saturated and unsaturated phospholipids that have very different phase-transition temperatures, e.g., DPPC and POPC (59,60). Furthermore, cholesterol can be added to these systems, which induces a condensing effect and accentuates the domain separation (61). It is postulated that the presence of domains, altering membrane fluidity and creating regions of lower packing density, may play a role in the penetration of ROS into the membrane. For completion, Fig. 1, *c* and *d* show that these mixed monolayers, i.e., of 1:1 POPC and DPPC or 1:1:1 POPC, DPPC, and cholesterol, are also stable when H_2O_2 is added.

Oxidation by other ROS: Fenton's reagent

Although H_2O_2 on its own is not capable of in situ oxidation of membrane lipids, other ROS, known to possess smaller energy barriers to bilayer penetration, can be significantly more reactive, e.g., radical species such as OH and HO_2 (57). Furthermore, these two highly reactive oxygen-containing radicals are generated in Fenton's reaction and hence are present under physiological conditions.

Fig. 3 *a* shows the temporal variation in surface pressure of a POPC monolayer after injecting 100 μL of H_2O_2 into the sub-phase containing 10 mg dm^{-3} (37 μM) $FeSO_4$. This concentration of $FeSO_4$ is at the lower end of the range used previously to assess the role of Fenton's reagent in oxidation (29,30,32–34). In contrast to Fig. 2 *b*, injection of H_2O_2 into the sub-phase now results in a noticeable drop in pressure, before rising slowly to equilibrate at a lower level after a period of ≈ 2 h.

This change in pressure for POPC on injecting H_2O_2 (Fig. 3 *a*) might suggest that, in a biological environment, H_2O_2 in combination with ferrous salts can result in oxidation to the alkyl tailgroups as found for oxidation by ozone (Fig. 2 *a*). However, the change in surface pressure caused by Fenton oxidation is reversed to that caused by ozonolysis, suggesting that the mechanism, and by implication the products, differ significantly. This is supported by the result shown in Fig. 3 *b*, where the effect of Fenton's reagent on the surface pressure of a DPPC film matches that of POPC, even though DPPC is fully saturated and inert to ozonolysis.

Clearly, the ROS produced by the Fenton reaction are oxidizing the lipids in a different way to oxidation by O_3. Furthermore, given that the response to Fenton's reagent for the unsaturated POPC and fully saturated DPPC is the same, it suggests that the oxidation by HO_2 and/or OH occurs independently of whether the lipid is saturated or not, and thus implies that the tailgroups have little impact on the oxidation. This is not surprising given the reactivity of the two radical species produced in the Fenton reaction and their high energy barrier for penetrating the hydrophobic region of a lipid bilayer, suggesting that reaction of the headgroup is more likely (57). This aligns with previous reactive density functional theory (DFT) simulations which concluded that oxidation through these ROS occurs primarily via the headgroup, and mainly by the OH species (62). This is entirely in accordance with the experimental observations in Fig. 3 because both POPC and DPPC have the same headgroup.

Given that the surface pressure profiles in Fig. 3, along with previous investigations, suggest that the oxidation occurs to the lipid headgroup and that the unsaturation in POPC plays no role, DPPC was selected as the lipid for further study because of its stability in air and its preponderance in cell membranes. In subsequent studies, the concentrations of H_2O_2 and $FeSO_4$ were fixed at ≈ 2.8 μM and 100 mg dm^{-3} (≈ 370 μM), respectively. This H_2O_2

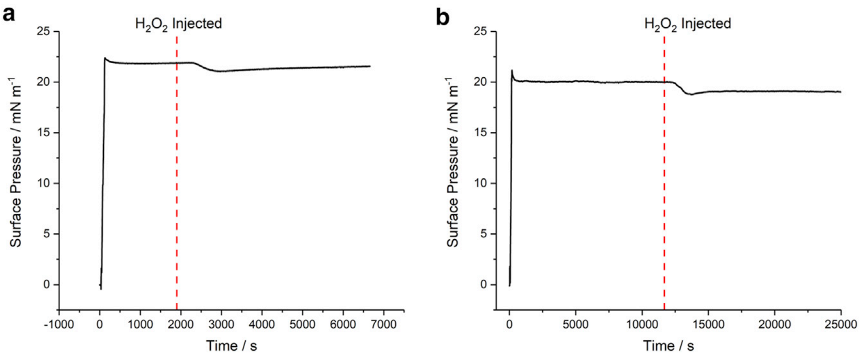

FIGURE 3 Temporal surface pressure behavior of a 20 mN m^{-1} lipid monolayer at the N_2-$FeSO_{4(aq)}$ interface (10 mg dm^{-3} $FeSO_4$) after injection of 100 μL of H_2O_2 into the sub-phase, for (*a*) POPC and (*b*) DPPC. To see this figure in color, go online.

concentration was chosen for its physiological relevance, as mentioned above (56), and the concentration of $FeSO_4$ was chosen to increase the detection sensitivity of any monolayer changes while remaining within the typical range used previously (29,30,32–34) and resulting in a moderate loss in surface pressure (aiming for $\approx$50% reduction). Although Fe^{2+} is supposed to be catalytic, and thus should not influence the extent of oxidation, this is not the case, due to the existence of other kinetic processes that are discussed in greater detail in the supporting material.

Dependence on pH

It is well known that Fenton's reagent is pH sensitive with the pH effect maximizing at an optimum pH of $\approx$3 (63–65). The pH effect is controlled by the ferrous and ferric ions. At too high a pH (i.e., low [H^+]), Fe^{3+} precipitates as ferric hydroxide or ferric oxide hydroxide, thus reducing the catalytic ability of the iron salts to convert H_2O_2 into HO_2 and OH. Furthermore, OH radicals show a lower redox potential at high pH, lowering their effectiveness as oxidants. At too low a pH (high [H^+]), the OH radicals can react with excess H^+ and their lower concentration yields less lipid oxidation. Additionally, at low pH, $[Fe(H_2O)_6]^{2+}$ becomes more stable in solution, reducing the availability of ferrous ions to produce ROS radicals. The results presented so far were recorded with an unadjusted sub-phase pH of $\approx$5.5 determined mainly by the dissolution of atmospheric CO_2. As a result, the reaction conditions used were far from generating optimum ROS.

The effect of pH on the oxidation of the membrane is illustrated in Fig. 4. Each point represents the observed (final) pressure drop recorded by temporally monitoring the surface pressure after the injection of H_2O_2, as in Fig. 3, as the sub-phase pH was adjusted incrementally using H_2SO_4 or NaOH. The approximate pH thresholds where ferric and ferrous hydroxide precipitate are indicated. Beyond the ferrous hydroxide precipitation limit at pH $\approx$8.5 no dissolved ferrous salts remain, so oxidation ceases. Across the physiological pH range (indicated in *pink*) there is also little variation in pH dependence until the threshold for ferric hydroxide precipitation is reached (at pH $\approx$3.5), beyond which the drop in pressure increases. It should be noted that, although the sub-phase pH affected the magnitude of the pressure drop, the temporal profile of surface pressure was qualitatively equivalent across the whole pH range (except above $\approx$8.5 where no changes were observed). This shows that the precipitation of the ferric hydroxide bears no significance to the observed changes in surface pressure other than preventing the regeneration of Fe^{2+}.

Although the catalytic behavior of Fenton's reagent maximizes at a pH $\approx$3, lower than that found in physiological environments, the Fenton oxidation can still make a significant contribution to the oxidation of cell membranes. It has previously been reported that immobilized heterogeneous ferrous/ferric catalysts, or those which are stabilized through chelating agents (strongly bound ligands), can extend the effective pH range of Fenton's reaction (67). Although heterogeneous iron catalysts bear no resemblance to physiological environments, the presence of ligands to bind the native iron ions should be expected, and thus is likely to prevent any precipitation and promote the catalytic behavior in the generation of ROS.

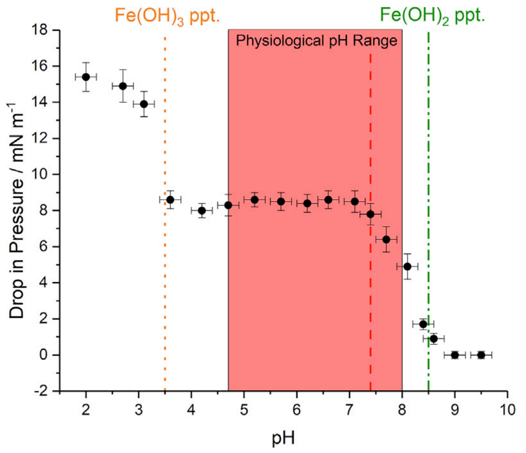

FIGURE 4 Drop in surface pressure of a DPPC monolayer initially formed at 20 mN m^{-1} at the air-$FeSO_{4(aq)}$ interface after the injection of H_2O_2, as a function of sub-phase pH. Also shown is the physiological pH range (in *pink*) and the homoeostatic pH in the body (*red dashed line*)(66), and the pHs when ferric (*orange dotted line*) and ferrous (*green dot-dashed line*) hydroxide precipitate. Uncertainties were calculated based on the pH measurement accuracy and variation in recorded pressure drops. To see this figure in color, go online.

Temporal Π-A isotherms during oxidation

The temporal characteristics of the oxidation are shown in Fig. 5, where isotherms recorded every 20 min after the injection of H_2O_2 are plotted. The reduction in surface pressure observed previously at a fixed trough area is confirmed,

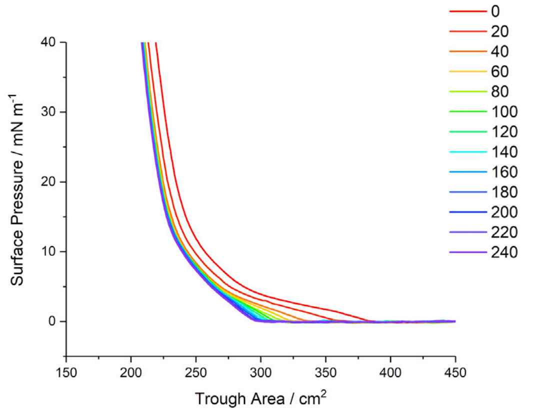

FIGURE 5 Π-A isotherms of a DPPC monolayer recorded at 20-min intervals after the injection of H_2O_2 into the sub-phase containing $FeSO_{4(aq)}$. To see this figure in color, go online.

but it is also clear that there are further changes to the isotherm in addition to a simple shift to lower areas. First, the liquid-expanded (LE)-liquid-condensed (LC) equilibrium profile that is characteristic of DPPC isotherms (68) is significantly diminished, suggesting a change in the intermolecular interactions within the monolayer. Furthermore, the compressibility of the film (indicated by the gradient in the isotherm) at high surface pressures reduces (observed as steeper rises in surface pressure with increasing time). These observed variations to the isotherm behavior of the film indicate significant structural alterations to the monolayer in response to ROS.

Dependence on initial surface pressure

It is known that the average molecular area available for lipids in a leaflet critically affects its properties (69). More densely packed leaflet lipids are less mobile, generally corresponding to an LC phase, and present a higher energy barrier for transport through the bilayer(70). It is expected that lipids at different surface pressures will therefore have different responses to oxidation due to the possible conformations that they can potentially adopt. The effect of different initial pressure on lipid oxidation is shown in Fig. 6 *a*, which plots the proportional (final) drop in pressure for initial pressures up to 50 mN m^{-1}.

Owing to the non-linearity of the surface pressure isotherm, particularly in the LE-LC equilibrium region, the change in surface pressure resulting from oxidation for different initial pressures is not expected to be constant or proportional to the initial pressure. Instead, a comparison can be made with the changes predicted from the surface pressure isotherms shown in Fig. 5. These are plotted alongside the observed changes in Fig. 6 *a*. Given that the changes in isotherms were measured with the monolayer exposed to ROS at 20 mN m^{-1}, any discrepancy between the predicted values and those observed for each initial pressure indicate a change in resistance to oxidation. These differences are emphasized in Fig. 6 *b*, which shows that the lower surface pressures ($<$ 20 mN m^{-1}) are more susceptible to oxidative changes as anticipated for less densely packed monolayers. Interestingly, higher surface pressures ($>$20 mN m^{-1}) are less susceptible but only up to $\approx$30 mN m^{-1}, after which their response to oxidation appears to increase. This is counterintuitive for densely packed films, as the higher density would be expected to provide better protection against ROS. Thus, the non-monotonic nature of these proportional pressure drops suggests there are multiple factors influencing the monolayer response, one possibility being a change in conformation at these higher surface pressures leading to greater oxidative stress. These results are particularly relevant from a physiological perspective, since the surface pressure of cell membranes is known to be $\approx$25–30 mN m^{-1} (71–78), hence optimizing the defense against oxidative changes.

SFG spectroscopy in the frequency and temporal domains

To characterize any structural or conformational changes in the monolayer in greater detail, SFG lipid spectra of the air-water interface were recorded as oxidation occurred. Fig. 7 shows spectra in the C-H (2800–3000 cm^{-1}) and C=O (1600–1800 cm^{-1}) stretching regions recorded before and 4 h after the injection of H_2O_2 into the sub-phase. The spectra clearly show an increase in intensity after 4 h both in the SSP and PPP polarization combinations for the main C-H bands, as well as in the SSP polarization in the carbonyl stretching region at $\approx$1730 cm^{-1}.

The orientation expected for lipids in well-packed monolayers has their tailgroups nearly normal to the interface; thus, their carbonyl groups will be nearly parallel to the interface and hence SFG-inactive. This is confirmed by the very low SFG intensities observed for the C=O stretching resonances at 1730 cm^{-1}, which are two orders of magnitude weaker than those of the methyl C-H resonances (although this decreased intensity will also be contributed to by the lower output power of the OPO at these frequencies cf. the C-H stretching region). The observed increase in the C=O resonance intensity after 4 h, therefore, means that the transition dipole now has a greater z-component, pointing to a greater tilt angle for the lipid tailgroup upon oxidation. This is accompanied by a marked shift in the C-H intensity

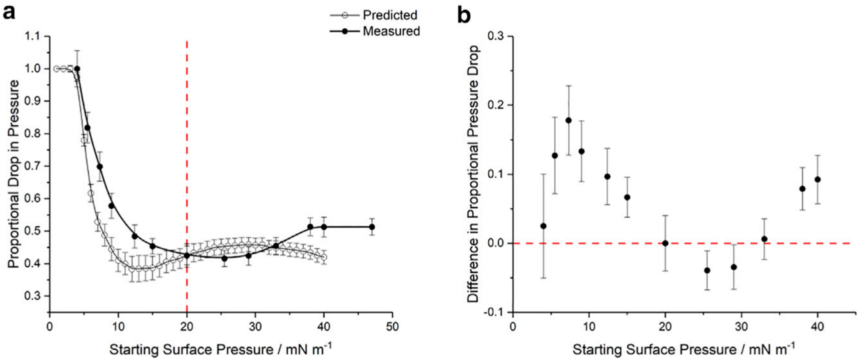

FIGURE 6 The effect of initial lipid pressure on the extent of Fenton oxidation. (*a*) The proportional drop in the surface pressure of a DPPC monolayer as a function of the initial pressure with both the measured values (*solid circles*) and those predicted from the changes to the isotherms in Fig. 5 (*open circles*). (*b*) The difference in proportional pressure drops shown in (*a*) as measured minus predicted. Uncertainties in the predicted values arise from the variation in isotherms (Fig. 5) and those in the measured values source from the variation in recorded pressure drops at each starting pressure. To see this figure in color, go online.

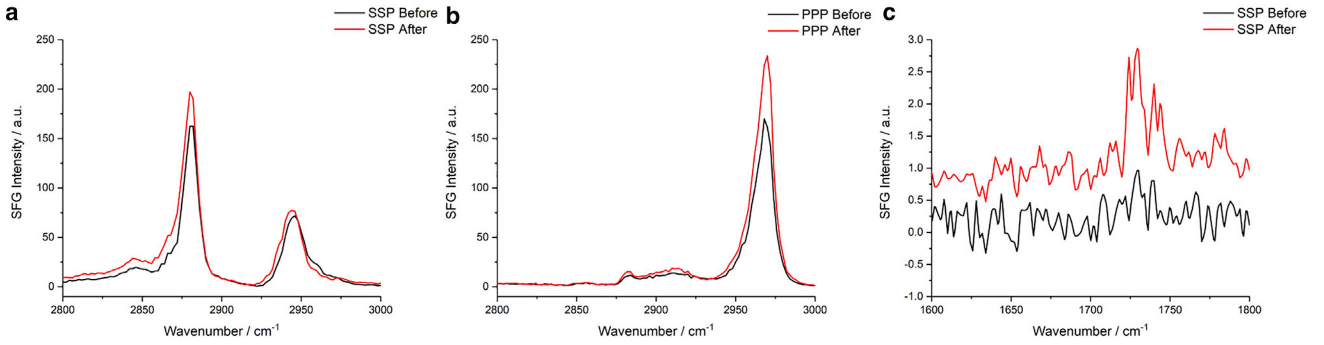

FIGURE 7 SFG spectra of a DPPC monolayer at the air-$FeSO_{4(aq)}$ interface before and 4 h after the injection of H_2O_2 into the sub-phase showing (*a*) SSP and (*b*) PPP spectra in the C-H stretching region, and (*c*) SSP spectra in the carbonyl stretching region, offset by 0.5 units for clarity. To see this figure in color, go online.

ratios showing that there have been structural changes to the monolayer. These structural changes can be quantified by fitting the spectra using the SFG equation, with the resulting spectral fits and underlying data points presented in Fig. 8. The conformational SFG ratios based on the d^+ (2849 cm^{-1}, SSP), r^+ (2881 cm^{-1}, SSP), and r^-_{OP} (2970 cm^{-1}, PPP) bands, deduced from the fitted spectra, are given in Table 1. There has been an increase in the d^+/r^+ ratio and a corresponding decrease in the r^+/r^-_{OP} ratio due to oxidation. The increase in the former ratio suggests increased conformational disorder in the alkyl tails of the lipid while the decrease in the latter ratio suggests an increase in the average methyl tilt angle (51). (This result correlates with the increase in the intensity of the carbonyl stretching resonances.) Both observations point to alkyl tails that have greater conformational freedom, causing a greater deviation from the all-*trans* conformation and an increase in the tilt angle.

An interesting contradiction arises from the observation of an increase in the r^+ resonance intensity in the SSP spectra, which is contrary to what is predicted for an increase in the average tilt angle. The r^+ resonance intensity should decrease for a monolayer at the air-water interface when the tilt angle increases. Additionally, the increased conformational disorder would be expected to further decrease all methyl contributions. This suggests that other factors must be considered to explain the increase in the r^+ resonance intensity. Some possibilities include:

1) The presence of a broad resonant or non-resonant band constructively interfering with the r^+ band

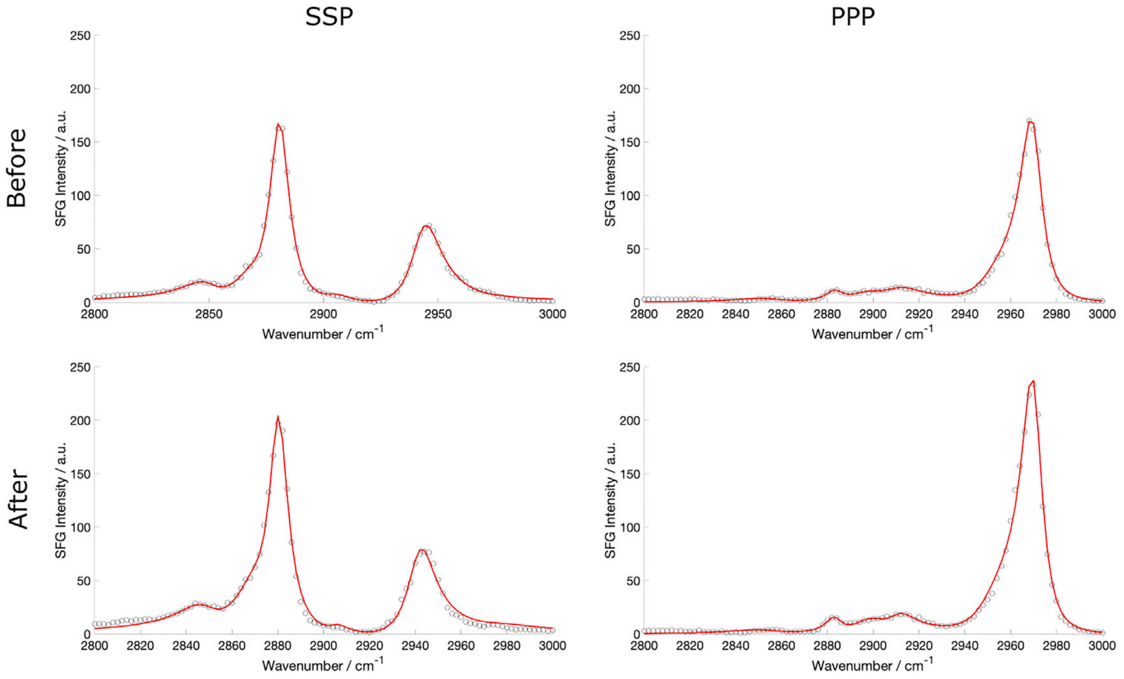

FIGURE 8 Fits of the experimental SFG spectra shown in Fig. 7 *a* and *b*. To see this figure in color, go online.

TABLE 1 Calculated ratios of susceptibility components deduced from the fits to the spectra in Fig. 8

Ratio of χ components	Before	After
$\left\|\frac{\chi_{SSP}(d^+)}{\chi_{SSP}(r^+)}\right\|$	0.178 ± 0.011	0.199 ± 0.008
$\left\|\frac{\chi_{SSP}(r^+)}{\chi_{PPP}(r_{OP}^-)}\right\|$	1.12 ± 0.04	0.75 ± 0.05

The observed changes, although relatively small, were confirmed for more than 10 independent measurements and can also be seen in the temporal SFG analysis.

2) A change in the lipid surface density, N
3) A change in Fresnel factors
4) A change in the depolarization derivative ratio, r
5) A non-isotropic surface

These possible explanations are now considered in greater detail.

A change in either the non-resonant contribution or other broad resonant contributions could combine in-phase with the r^+ band, increasing its intensity. This, however, is unlikely since, although there is a slight increase in the baseline in the ≈2800–2900 cm^{-1} region, it is not significant enough. Furthermore, the PPP spectra in Fig. 7 *b* clearly show an increase in both r^+ and r^-_{OP} bands despite no underlying broad resonances or non-resonant contribution. Although the r^+ intensity in PPP does not change monotonically with tilt angle as in SSP, for a fairly "upright" monolayer it would also be expected to decrease on increasing the tilt angle, contrary to what is observed. Overall, the spectral fitting did not yield a significant non-resonant contribution in any of the spectra, thus demonstrating that this cannot be the source of the observed intensity variations.

The average number density, $\langle N \rangle$, cannot change because the SFG experiments were recorded in a Langmuir trough with a static barrier and so the area available for the monolayer remains unchanged throughout the oxidation process. Nonetheless, SFG from a dielectric depends on the square of the number density, $\langle N^2 \rangle$, which can change. Specifically, a transition from a uniform monolayer to one with domains could generate tightly packed regions as well as a less dense phase. However, this would result in the SFG spectra being dominated by contributions from the tightly packed domains, which are the least expected to show an increase in the tilt angle and greater conformational disorder.

A change to the Fresnel factors is considered unlikely to arise solely from the oxidation of a monolayer at the air-water interface. This would require a significant change in the refractive index of the monolayer, which is highly improbable given the observation that the alkyl tail structure appears to be largely unchanged.

Furthermore, in practice, the SFG spectra are dominated by the terminal methyl groups from both alkyl tails, and it is difficult to envisage the oxidation resulting in a change in the local environment to such an extent that the depolarization ratio for these bands is significantly altered.

Finally, it is almost universally assumed in analyzing SFG spectra that the surface is isotropic. A violation of this symmetry would result in significant alterations to the expected intensity variations. Consequently, a break in this symmetry before and/or after oxidation would influence the observed changes. Although in-plane isotropy is expected at the air-water interface due to the isotropy of both bulk phases, the unidirectional compression of the monolayer could potentially introduce anisotropy in the surface. This possibility has been raised previously and has been demonstrated when casting monolayers onto solid supports (79). However, SFG spectra of the C-H stretching region in the SPP and PSP polarization combinations showed no intensity, indicating minimal if any anisotropy (80).

The most probable explanation of the observed intensity increase is that it arises from a combination of molecular structural/conformational changes in addition to small changes contributed by a subset of the above factors. Further work is required to isolate the source of these unexpected intensity variations.

A further notable observation from the spectra in Fig. 7 comes from the intensity changes at ≈2910 and ≈2960 cm^{-1} in the SSP polarization combination (Fig. 7 *a*), with the former being almost constant and the latter showing a clear decrease on oxidation, in contrast to the overall increase in intensity for other bands. Previous work on d_{62}-DPPC showed that the (protonated) choline moiety in the headgroup has two bands at these frequencies (with the latter arising from the symmetric methyl stretch, being the stronger) that are prominent in SFG spectra in the SSP polarization combination (but only very weakly present in PPP) (81). SFG spectra at these frequencies will, typically, also be contributed to by antisymmetric resonances (e.g., d^- at ≈2910 cm^{-1} and r^-_{OP} at ≈2960 cm^{-1}), yet the polarization selection rules for almost upright monolayers dictate that these will only show strong contributions in the PPP spectra (as observed in Fig. 7 *b*). Nevertheless, for reasonably well-packed monolayers, a small r^-_{OP} band is commonly found in SSP spectra within this spectral region. However, an increase in the tilt angle would be expected to cause an increase in the r^-_{OP} band in SSP (along with the decrease in r^+ mentioned above) (51). Hence, the observed changes to SSP spectra in these regions are likely to be dominated by changes to the choline moieties. This suggests either a change in conformation of the choline group such that their dipole moments point further away from the surface normal, thus reducing their SFG intensity, or a reduction in their surface density arising from chemical attack by the ROS.

SFG temporal analysis

To shed more light on the temporal changes in lipid structure that occur on oxidation lower resolution, sequential SFG

spectra were recorded after the injection of H_2O_2. Fig. 9 shows SFG spectra recorded alternately in SSP and PPP polarizations at 12-min intervals for $\approx$5 h. The higher temporal resolution data in Fig. 9 show that the spectral changes do not take place incrementally (as might be inferred from Fig. 7) but occur abruptly, with significant spectral overlap existing for spectra either side of a sharp transition at $\approx$30 min after the injection of H_2O_2.

As in Fig. 7 *a*, Fig. 9 *a* also shows a reduction in intensity at $\approx$2960 cm^{-1} and a constant or slight decrease in intensity at $\approx$2910 cm^{-1} on oxidation, despite the overall increase in intensity for the other bands. This further points to changes to the choline moiety within the headgroup as mentioned above.

The change in the temporal response at the specific frequencies of known C-H stretching and carbonyl group resonances, and the corresponding change in surface pressure, is more clearly apparent in Fig. 10. After a small initial increase in SFG intensity, the C-H bands show a pronounced dip and then subsequently slowly increase but to different extents. This implies that the oxidation mechanism comprises several kinetic steps, each resulting in conformational changes to the monolayer. As similar behavior is observed for all bands, it is also suggested that the observed intensity variations arise from a convolution of these conformational changes that affect both the absolute and relative intensities, with changes arising from other, unknown factors, potentially including a subset of those mentioned above (giving rise to the unexpected increase in the r^+ band in the SSP spectra). Overall, however, there are clear conformational changes which indicate that the oxidation results in a less ordered monolayer with increased tilt angles.

Chemical changes from FTIR spectroscopy

Although the surface tensiometry and SFG results above indicate that structural and conformational changes are occurring during oxidation, they provide little detail about the specific chemical changes in the monolayer. To provide answers to these questions, ATR and IRRAS spectra of the monolayer were recorded using FTIR spectroscopy.

Multi-reflection ATR

To compensate for the generally low IR absorption of monolayer thick films, multi-reflection ATR spectroscopy was used to increase the effective path length. Although this significantly improves both the signal strength and sensitivity, temporal studies require sequential casting of monolayers, which is time consuming. A further consideration concerns the oxidation of the Ge ATR crystal surface. The GeO_2 product absorbs strongly below 900 cm^{-1} and is slightly water soluble, making accurate background subtraction difficult. For this reason, only monolayer spectra >900 cm^{-1} were investigated. It should also be noted that some residual water vapor interference also occurs in the d_{75}-DPPC spectra owing to the much smaller extinction coefficients of the C-D bands (cf. C-H). Consequently, normalization renders signal noise more pronounced.

Fig. 11, *a* and *b* show spectra of DPPC and d_{75}-DPPC monolayer films, respectively, cast onto a 25-reflection germanium ATR crystal via LB deposition before, and more than 4 h after, the injection of H_2O_2.

Comparing the initial and final spectra in Fig. 11, it is clear that many features remain largely unchanged, e.g., the bands >$\approx$1300 cm^{-1}. There are, however, significant changes in the lower frequency region from $\approx$900 to 1300 cm^{-1}. On oxidation, DPPC (Fig. 11 *a*) shows a decrease in the bands centered at 1020, 1060, and 1242 cm^{-1} and an increase in intensity over the $\approx$1070–1200 cm^{-1} region. For d_{75}-DPPC, however, deuteration shifts the C-H backbone modes to the 1000–1300 cm^{-1} region, causing much greater overlap with the headgroup bands. Nevertheless, d_{75}-DPPC (Fig. 11 *b*) still shows a change in band structure in this spectral range, with a large increase in intensity at 1000–1200 cm^{-1}, a notable reduction in the 1242 cm^{-1} band, and apparent reductions in the 1020 and 1060 cm^{-1} bands, which appear as weakened shoulders in the "after" spectrum.

The literature assignments of the bands observed for DPPC and d_{75}-DPPC are given in Table 2 (82–87). These data in conjunction with Fig. 11 show that oxidation results in a lowering of the intensity of the bands assigned to PO_2-containing moieties. Furthermore, the increase in intensity

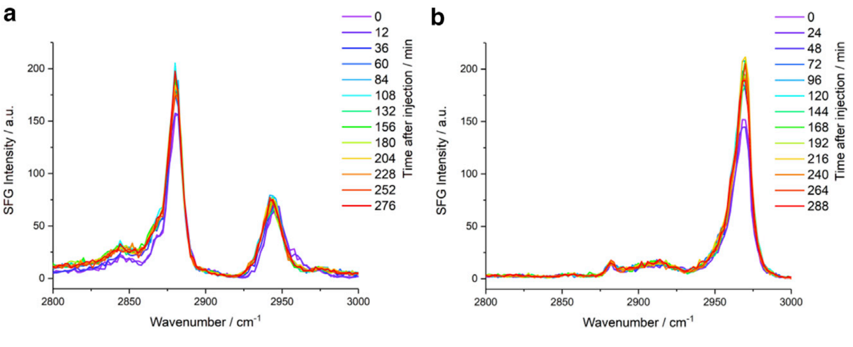

FIGURE 9 SFG spectra in the C-H stretching region of a DPPC monolayer at the air-$FeSO_{4(aq)}$ interface in the (*a*) SSP and (*b*) PPP polarization combinations recorded alternately every 12 min over a period of 5 h after the injection of H_2O_2 into the sub-phase. To see this figure in color, go online.

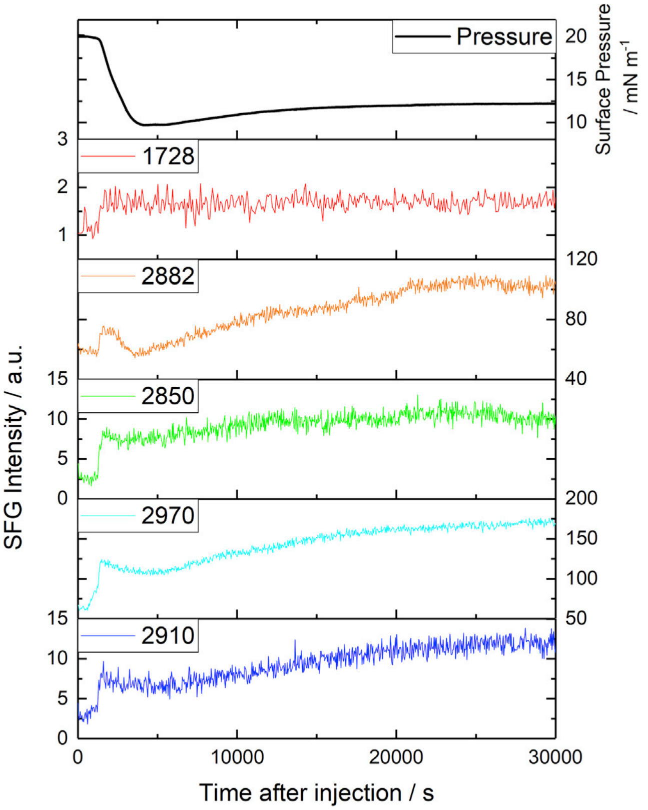

FIGURE 10 Temporal changes in specific SFG resonances of a DPPC monolayer at the air-$FeSO_{4(aq)}$ interface after injection of H_2O_2 into the sub-phase. Surface pressure (*top trace*) and SFG intensities at resonances corresponding to specific bands of DPPC: 1728 cm^{-1} (SSP) ester carbonyl, 2882 cm^{-1} (SSP) r^+, 2850 cm^{-1} (SSP) d^+, 2970 cm^{-1} (PPP) r^-_{OP}, and 2910 cm^{-1} (PPP) d^-. To see this figure in color, go online.

between $\approx$1000 and 1200 cm^{-1} indicates the addition of O-containing moieties, a consequence of oxidation producing C-O functionality that contributes to this spectral region (88–90).

Other than the significant spectral changes discussed above, the ATR spectra in Fig. 11 also show smaller changes to other bands, including the carbonyl band at 1736 cm^{-1} and the trimethyl ammonium (choline) band at 970 cm^{-1}. Additionally, the antisymmetric methyl band appears to decrease in intensity (cf. the antisymmetric methylene band). This could suggest there is an increase in the methylene/methyl ratio, which is not anticipated to occur as a result of oxidation. In contrast, the symmetric methyl band appears to show a slight increase in intensity, directly contradicting this conclusion. (This is not easy to pick out in the protonated DPPC spectra owing to the reversed order in the position of the methyl and methylene modes cf. perdeuterated DPPC.) As discussed earlier, the choline moieties also give rise to intensity at $\approx$2960 cm^{-1} and thus can cause a reduction in intensity should their surface density reduce. This is a likely contribution to the observed intensity loss at 2960 cm^{-1}, as the 970 cm^{-1} band associated with choline also shows a slight decrease. Unlike the SSP SFG spectra discussed earlier, however, IR spectra will show greater contributions from the antisymmetric methyl modes of the tailgroups (both in-plane and out-of-plane). Therefore, the increase in methyl symmetric/antisymmetric ratio can also have significant contributions from conformational changes occurring in the monolayer. Owing to the different Fresnel factors for P- and S-polarized light, ATR generates unequal field strengths in the principal axes. Thus, a well-defined molecular orientation with groups that possess different dipole moment vectors can yield changing ratios with molecular orientation. An increase in symmetric/antisymmetric ratio can be shown to indicate an increase in tilt angle of the lipid tailgroups, consistent with the SFG analysis above.

Temporal IRRAS analysis

IRRAS, unlike ATR, can be performed at the air-water interface, enabling temporal study of the lipid oxidation. It does, however, yield significantly lower spectral intensities than ATR, and the presence of water in the spectrometer from the sub-phase introduces significant water vapor interference, which proves difficult to subtract.

IRRAS spectra in the 900–1300 and 2800–3000 cm^{-1} regions (omitting the 1300–1800 cm^{-1} region, already recorded by ATR, due to water vapor interference) are shown in Fig. 12, with spectra recorded every 6 min for 2 h after the injection of H_2O_2. The recorded spectra actually appear

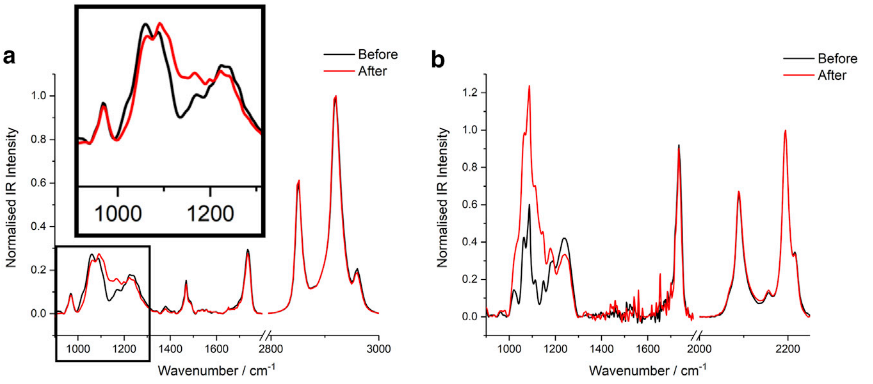

FIGURE 11 ATR-FTIR spectra of a 20 mN m^{-1} film of (*a*) DPPC and (*b*) d_{75}-DPPC monolayer LB-cast onto a 25-reflection Ge ATR crystal before and after (>4 h) injection of H_2O_2 into the sub-phase containing $FeSO_4$. Each spectrum represents the average of (*a*) 15 and (*b*) 4 independent measurements and have been normalized to the antisymmetric methylene band (d^-). To see this figure in color, go online.

TABLE 2 Literature assignments of the bands observed in the ATR spectra of DPPC and d_{75}-DPPC

Band/cm^{-1}	DPPC	d_{75}-DPPC	Assignment
970	•		$N^+(CH_3)_3$ asym.
1020	•	•	C-O-PO_2^-
1060	•	•	C-O-PO_2^-
1088	•	•	PO_2 sym./CD_2 bend
1110		•	CD_2 bend
1146		•	CD_2 bend
1178	•	•	CO-O
1200	•		CH_2 bend
1225	•		CH_2 bend
1242	•	•	PO_2 asym.
1262	•		CH_2 bend
1283	•		CH_2 bend
1342	•		CH_3 bend
1380	•		CH_3 bend
1417	•		CH_3 bend
1469	•		CH_2 bend
1488	•		N-CH_3 bend
1736	•	•	C=O str.
2070		•	CD_3 sym.
2090		•	CD_2 sym.
2156		•	CD_2 FR
2195		•	CD_2 asym.
2216		•	CD_3 asym.
2851	•		CH_2 sym.
2879	•		CH_3 sym.
2900	•		CH_2 FR
2919	•		CH_2 asym.
2960	•		CH_3 asym.

as dips but are converted to positive-going peaks after normalization by division by the CH_2 antisymmetric mode intensity. Spectra could only be recorded for 2 h because of increasing contributions from water vapor interference.

As for the ATR spectra, the IRRAS spectra (Fig. 12) show clear changes on oxidation. First, there is a reduction in the 1242 cm^{-1} antisymmetric PO_2 band as well as apparent reductions in the 1060 cm^{-1} band (which appears as a reduced shoulder) and the trimethyl ammonium (choline) band at 970 cm^{-1}. These correspond well with the findings from the ATR spectra and suggest significant changes to the lipid headgroup. Furthermore, there is a large-intensity increase in the 1050–1150 cm^{-1} region, as observed in the ATR spectra, which points to an increase in C-O moieties. The apparently negative band at $\approx$1220 cm^{-1} (as well as the weaker one at $\approx$920 cm^{-1}) is from the $CHCl_3$ lipid solvent, which remains in minuscule amounts.

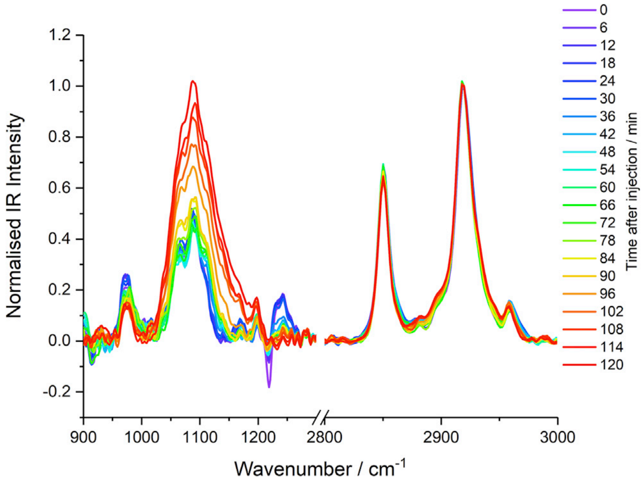

FIGURE 12 IRRAS spectra of a DPPC monolayer at the air-$FeSO_4$(aq) interface in the 900–1300 and 2800–3000 cm^{-1} regions recorded every 6 min after the injection of H_2O_2 into the sub-phase. The negative features at $\approx$920 and $\approx$1220 cm^{-1} are due to residual $CHCl_3$ solvent. To see this figure in color, go online.

There are also subtle changes in the C-H stretching region, particularly to the r^- band (from both in-plane and out-of-plane modes), which appears to slightly sharpen, shift to a lower frequency, and reduce in intensity. A similar change in the r^+ band also occurs, except that it shifts in the other direction (to higher frequency) and increases in intensity on oxidation. These observations align well with the observations noted in the d_{75}-DPPC ATR spectra (Fig. 11 *b*) which also showed this relative intensity change for the two methyl bands. As mentioned earlier, this indicates a conformational change in the monolayer rather than any significant change to the structure of the alkyl chain and can also suggest a loss in choline surface density, chiming with the reduction in the 970 cm^{-1} band. In contrast, the slight changes in band widths and band centers for the two methyl modes are indicative of minor alterations to their chemical environment which likely arise from changes to the intermolecular interactions between the tailgroups, consistent with the changes to surface pressure isotherms discussed previously.

One further notable change in the C-H band structure arises from the band center of the antisymmetric methylene mode (d^-) centered at $\approx$2920 cm^{-1}. The position of this band is commonly used as an indicator of the conformational order (91) as it is influenced by the intermolecular environment (i.e., the molecular packing). Band-center analysis of eight independent sets of IR spectra show a slight shift in the band position to higher frequency, as indicated in Table 3. This indicates a decrease in conformational order, consistent with the changes in d^+/r^+ ratio (Table 1) in the SFG spectra discussed earlier.

The temporal changes in intensity in the fingerprint region are found to be different for the individual bands. Fig. 13 shows the integrated band intensity for the asym. $N(CH_3)_3^+$ band at 970 cm^{-1} (*top*), the C-O stretching region

TABLE 3 Band-center analysis of the antisymmetric methylene mode (d^-), averaged over eight independent measurements

Band-center analysis	Value/cm^{-1}
Band-center before oxidation	2918.9 $\pm$ 0.8
Band-center after oxidation	2919.8 $\pm$ 1.0
Avg. difference in band centers	0.9 $\pm$ 0.7

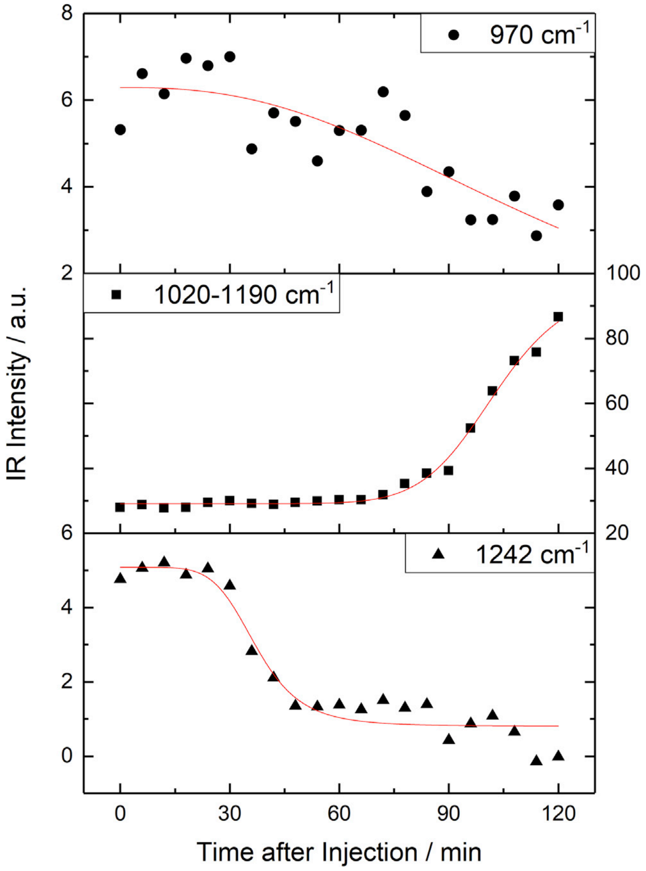

FIGURE 13 Temporal analysis of the IRRAS spectra in Fig. 12 showing the variation in three selected bands after the injection of H_2O_2: the asym. $N(CH_3)_3^+$ band at 970 cm^{-1} (*top*), the C-O stretching region between 1020 and 1190 cm^{-1} (*middle*), and the asym. PO_2 band at 1242 cm^{-1} (*bottom*). Intensities are from integration of the band in the normalized spectra (relative to the asym. CH_2 band). Each temporal trace has been fitted with a logistic sigmoidal function for qualitative analysis. To see this figure in color, go online.

between 1020 and 1190 cm^{-1} (*middle*), and the asym. PO_2 band at 1242 cm^{-1} (*bottom*). The temporal change of each band has been fitted with a logistic sigmoidal function as a guide to the eye. First, the $N(CH_3)_3^+$ band from the choline headgroup (970 cm^{-1}) appears to show an initial delay period of $\approx$30 min after the injection of H_2O_2 and then subsequently decrease. A similar behavior is observed for the PO_2 band (1242 cm^{-1}), with a delay period of $\approx$30 min followed by a much sharper intensity decrease to reach a fairly constant level over the next 15–30 min (45–60 min after injection). The temporal profiles of both these bands are in marked contrast to the 1020–1190 cm^{-1} feature where its intensity remains unchanged for $\approx$60 min after the injection of H_2O_2, after which it strongly increases.

These temporal variations in the fingerprint region strongly suggest that the oxidation occurs via a multi-step mechanism and are consistent with the temporal SFG analysis that indicated multiple kinetic processes. Specifically, there appears to be an initial delay period after the injection of H_2O_2. This could either be due to slow conversion kinetics of H_2O_2 into OH and HO_2 or be diffusion-limited within the sub-phase. After this initial delay period, the primary center of attack of the newly generated ROS radicals is the headgroup of the lipid, causing a reduction in the choline and phosphate band intensities. Subsequently, the oxidants appear to add significant C-O-containing groups to the lipid. Interestingly these C-O groups do not appear to originate from the alkyl tails since there are only very minor changes in the C-H stretching region, suggesting no significant loss in CH_2 groups. These observations chime well with previous reactive DFT simulations which led to the conclusion that the most significant reaction of the OH radicals was with the choline headgroup, with only minor pathways corresponding to attack of the choline or glycerol CH_2 backbones, leading to the appearance of C-O functionality (62). There is, however, one difference between the simulation results and the observations in this work concerning the phosphate group. The simulations all showed no loss of the phosphate group on oxidation, which contradicts the definite loss of intensity of the asym. PO_2 band, as shown in Fig. 13. A feasible hypothesis for the experimental observation is that the attack on the glycerol backbone of the headgroup can result in cleavage of phosphate moieties from the rest of the lipid. Some possible mechanistic pathways are shown in Fig. 14.

The mechanisms in Fig. 14 all involve H-abstraction by OH as the initial step, a common and well-established pathway for these ROS (92,93). Each of the mechanisms A to F presents the likely reaction following the H-atom abstraction of all available (labile) H atoms in the headgroup, starting at the choline group and then progressing down the headgroup to the hydrocarbon chain. Similar mechanisms have already been suggested based on the results of the reactive DFT simulations, specifically A, C, D, and F (62). Although pathway A was pinpointed as dominant, analysis of experimental mass spectroscopy results suggested that pathways D and F were the major ones corresponding to cleavage of one of the acyl chains, which would then result in expulsion of carboxyl radicals. This previous work also suggested that the resulting carboxyl radical from the cleaved chain expels CO_2, ultimately resulting in a long-chain alcohol via another reaction with OH. The IR spectral results, however, do not support this, as there is no significant loss in C=O intensity, and it is concluded that these mechanisms are either minor or that the loss of CO_2 from the headgroup is very slow.

Mechanisms A, B, C, and E all involve cleavage of the headgroup, with each one removing the choline amino group but only mechanism E expelling the phosphate group. Based on the IRRAS analysis presented above (Fig. 12), these four mechanisms are suggested to dominate, particularly since there is a clear loss in both the asym. $N(CH_3)_3^+$ and PO_2 bands on oxidation. The high reactivity of OH radicals means that many different pathways may be active and lead to the loss of phosphate and choline groups

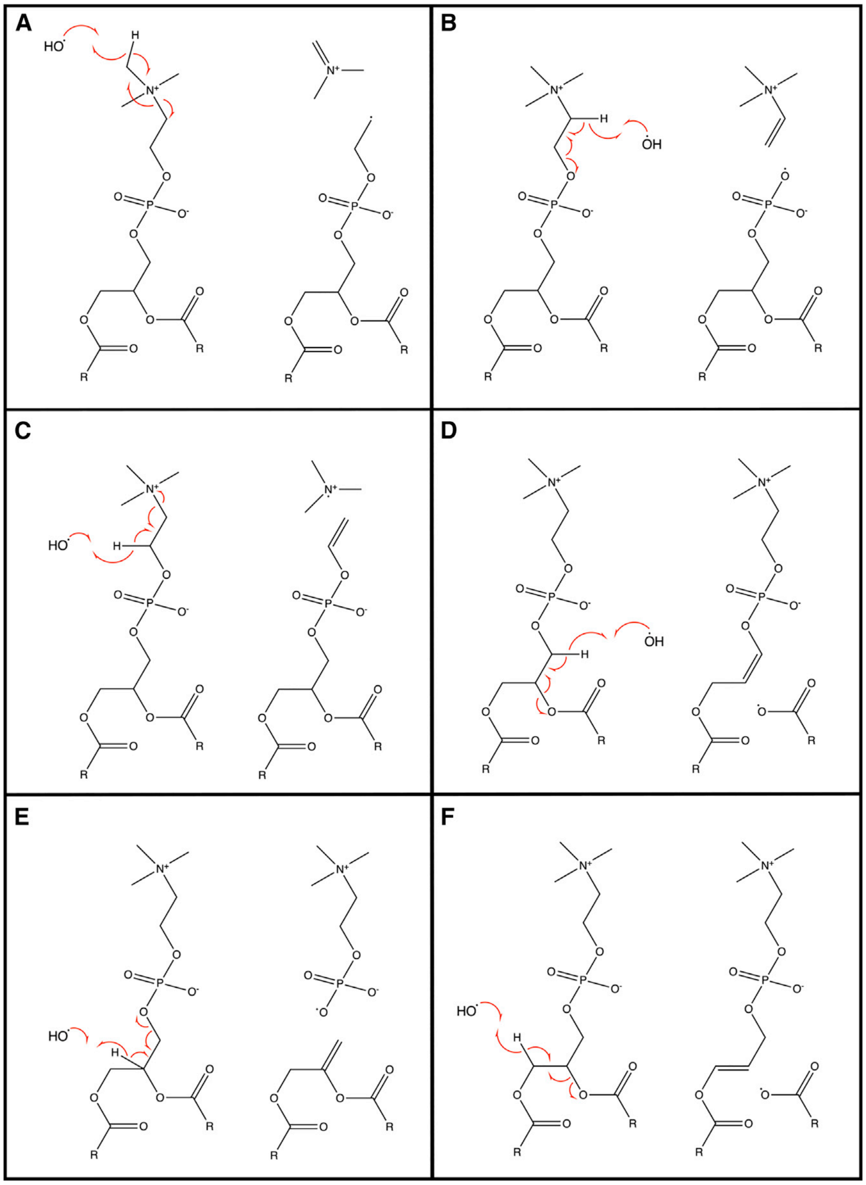

FIGURE 14 Possible mechanistic pathways for the oxidation of DPPC by OH radicals. To see this figure in color, go online.

in subsequent steps rather than in the initial reactions. Furthermore, some of these mechanisms lead to the formation of centers of unsaturation. These too are known to be centers of oxidation, ultimately adding C-O functionality. This provides an explanation for the observed increase in C-O intensity in the IRRAS spectra after the initial loss of headgroup spectra.

As there is strong evidence for cleavage of certain moieties in the headgroup and potentially one of the acyl tails, it is important to consider the impact that these molecules may have on the surface number density and molecular structure of the remaining (oxidized) lipid. Regarding the loss of small, charged molecules arising from the cleavage of headgroup functionality, i.e., choline- or phosphate-containing molecules, these are expected to be highly soluble in the sub-phase and thus have minimal impact on the remaining monolayer. By contrast, the cleavage of one acyl chain will essentially convert the di-chain lipid into two (different) single-chain lipids, both thus being strongly surface active. Therefore, the surface number density will in principle increase, but without any "real" change in molecular density at the interface. Instead, it is anticipated that this cleavage would allow for greater freedom of the two tails and may be a source of the observed reduction in surface pressure on oxidation.

CONCLUSION

A combination of surface tensiometry, SFG, ATR, and IRRAS have been used to elucidate the mechanism of

oxidation of membrane lipids by H_2O_2, particularly focusing on DPPC. Although H_2O_2 alone was shown to have no effect, in combination with Fe^{2+} ions it produces OH and HO_2 radicals through Fenton's reaction, which has significantly greater oxidative potency. On introducing these ROS in situ they were shown to cause substantial chemical changes specifically to the phospholipid headgroup, ultimately leading to a monolayer with greater disorder and higher tilt angles that possessed significantly different intermolecular interactions. The proposed mechanism of the oxidative attack involves loss of both the choline and phosphate moieties within the headgroup followed by the addition of C-O functionality, all occurring via processes with different speeds kinetically. These processes can occur in vivo owing to the presence of Fe^{2+} in biological systems and the relatively high physiological levels of H_2O_2 (as used in this investigation). Hence, the substantial membrane alteration and disruption reported in this study are also likely to be present in vivo and play a significant role in physiology, particularly with regard to oxidative stress and non-apoptotic or premature apoptotic cell death.

DATA AVAILABILITY

The data that support the findings of this study are available from the corresponding author upon reasonable request.

SUPPORTING MATERIAL

Supporting material can be found online at https://doi.org/10.1016/j.bpj.2022.10.040.

AUTHOR CONTRIBUTIONS

A.P.F. designed the research, performed the experiments, analyzed the data, and drafted the manuscript. M.T.L.C. oversaw the project, acquired the funding, and helped with the experimental design and interpretation of data. P.B.D. oversaw the project, helped with the experimental design and interpretation of data, and edited the manuscript.

ACKNOWLEDGMENTS

This research was funded, in whole or in part, by the Engineering and Physical Sciences Research Council (EPSRC) and Unilever Research under grant no. EP/R511870/1. For the purpose of Open Access, the author has applied a CC BY public copyright license to any Author Accepted Manuscript (AAM) version arising from this submission.

DECLARATION OF INTERESTS

The authors declare no competing interests.

REFERENCES

1. Cooper, G. M. 2000. The Cell: A Molecular Approach. Sunderland (MA): Sinauer Associates.
2. Gaschler, M. M., and B. R. Stockwell. 2017. Lipid peroxidation in cell death. *Biochem. Biophys. Res. Commun.* 482:419–425.
3. Tarr, M., and F. Samson. 1993. Oxygen Free Radicals in Tissue Damage, 1st ed. Birkhäuser, New York.
4. Szebeni, J., C. C. Winterbourn, and R. W. Carrell. 1984. Oxidative interactions between haemoglobin and membrane lipid. A liposome model. *Biochem. J.* 220:685–692.
5. Radi, R., J. S. Beckman, K. M. Bush, and B. A. Freeman. 1991. Peroxynitrite-induced membrane lipid peroxidation: the cytotoxic potential of superoxide and nitric oxide. *Arch. Biochem. Biophys.* 288:481–487.
6. Cejas, P., E. Casado, C. Belda-Iniesta, J. De-Castro, E. Espinosa, A. Redondo, M. Sereno, M. Á. García-Cabezas, J. A. F. Vara, A. Domínguez-Cáceres, R. Perona, and M. González-Barón. 2004. Implications of oxidative stress and cell membrane lipid peroxidation in human cancer. *Cancer Causes \& Control.* 15:707–719.
7. Fernandis, A. Z., and M. R. Wenk. 2007. Membrane lipids as signaling molecules. *Curr. Opin. Lipdology.* 18:121–128.
8. Bochkov, V. N., O. V Oskolkova, K. G. Birukov, A.-L. Levonen, C. J. Binder, and J. Stöckl. 2010. Generation and biological activities of oxidized phospholipids. *Antioxidants \& Redox Signal.* 12:1009–1059.
9. Hong, R., T. Y. Kang, C. A. Michels, and N. Gadura. 2012. Membrane lipid peroxidation in copper alloy-mediated contact killing of \textit {Escherichia coli. *Appl. Environ. Microbiol.* 78:1776–1784.
10. Phaniendra, A., D. B. Jestadi, and L. Periyasamy. 2015. Free radicals: properties, sources, targets, and their implication in various diseases. *Indian J. Clin. Biochem.* 30:11–26.
11. Attri, P., Y. H. Kim, D. H. Park, J. H. Park, Y. J. Hong, H. S. Uhm, K.-N. Kim, A. Fridman, and E. H. Choi. 2015. Generation mechanism of hydroxyl radical species and its lifetime prediction during the plasma-initiated ultraviolet (UV) photolysis. *Sci. Rep.* 5:9332.
12. Bacellar, I. O. L., and M. S. Baptista. 2019. Mechanisms of photosensitized lipid oxidation and membrane permeabilization. *ACS Omega.* 4:21636–21646.
13. Caetano, W., P. S. Haddad, R. Itri, D. Severino, V. C. Vieira, M. S. Baptista, A. P. Schröder, and C. M. Marques. 2007. Photo-induced destruction of giant vesicles in methylene blue solutions. *Langmuir.* 23:1307–1314.
14. Mertins, O., I. O. L. Bacellar, F. Thalmann, C. M. Marques, M. S. Baptista, and R. Itri. 2014. Physical damage on giant vesicles membrane as a result of methylene blue photoirradiation. *Biophys. J.* 106:162–171.
15. Schmidt, T. F., L. Caseli, O. N. Oliveira, and R. Itri. 2015. Binding of methylene blue onto Langmuir monolayers representing cell membranes may explain its efficiency as photosensitizer in photodynamic therapy. *Langmuir.* 31:4205–4212.
16. McDowell, M. S., A. Bakac, and J. H. Espenson. 1983. A convenient route to superoxide ion in aqueous solution. *Inorg. Chem.* 22:847–848.
17. Shahid, M., B. Pourrut, C. Dumat, M. Nadeem, M. Aslam, and E. Pinelli. 2014. Reviews of Environmental Contamination and Toxicology. Springer, New York, pp. 1–44.
18. Yu, Z., Q. Li, J. Wang, Y. Yu, Y. Wang, Q. Zhou, and P. Li. 2020. Reactive oxygen species-related nanoparticle toxicity in the biomedical field. *Nanoscale Res. Lett.* 15:115.
19. Fu, P. P., Q. Xia, H.-M. Hwang, P. C. Ray, and H. Yu. 2014. Mechanisms of nanotoxicity: generation of reactive oxygen species. *J. Food Drug Anal.* 22:64–75.
20. Li, M., J.-J. Yin, W. G. Wamer, and Y. M. Lo. 2014. Mechanistic characterization of titanium dioxide nanoparticle-induced toxicity using electron spin resonance. *J. Food Drug Anal.* 22:76–85.
21. Fenton, H. J. H. 1894. Oxidation of tartaric acid in presence of iron. *J. Chem. Soc. Trans.* 65:899–910.
22. Symons, M. C. R., and J. M. C. Gutteridge. 1998. Free Radicals and Iron: Chemistry, Biology, and Medicine, 1st ed. Oxford University Press, Oxford.
23. Burkitt, M. J. 2003. Chemical, biological and medical controversies surrounding the Fenton reaction. *Prog. React. Kinet. Mech.* 28:75–103.

24. Prousek, J. 2007. Fenton chemistry in biology and medicine. *Pure Appl. Chem.* 79:2325–2338.
25. Zhao, Z. 2019. Iron and oxidizing species in oxidative stress and alzheimer's disease. *Aging Med.* 2:82–87.
26. Thomas, C., M. M. Mackey, A. A. Diaz, and D. P. Cox. 2009. Hydroxyl radical is produced via the Fenton reaction in submitochondrial particles under oxidative stress: implications for diseases associated with iron accumulation. *Redox Rep.* 14:102–108.
27. Rifkind, J. M., J. G. Mohanty, and E. Nagababu. 2015. The pathophysiology of extracellular hemoglobin associated with enhanced oxidative reactions. *Front. Physiol.* 5:500.
28. Sadrzadeh, S. M., E. Graf, S. S. Panter, P. E. Hallaway, and J. W. Eaton. 1984. Hemoglobin. A biologic Fenton reagent. *J. Biol. Chem.* 259:14354–14356.
29. Gutteridge, J. M. 1984. Lipid peroxidation initiated by superoxide-dependent hydroxyl radicals using complexed iron and hydrogen peroxide. *FEBS Lett.* 172:245–249.
30. Zhang, D., C. Gong, J. Wang, C. Mu, W. Wang, and X. Zhang. 2021. Beyond lipid peroxidation: distinct mechanisms observed for POPC and POPG oxidation initiated by UV-enhanced Fenton reactions at the air–water interface. *J. Mass Spectrom.* 56, e4626.
31. Schaich, K. M., and D. C. Borg. 1988. Fenton reactions in lipid phases. *Lipids.* 23:570–579.
32. Rynkowska, A., J. Stepniak, and MKarbownik-Lewińska. 2020. Fenton reaction-induced oxidative damage to membrane lipids and protective effects of 17$β$-estradiol in porcine ovary and thyroid homogenates. *Int. J. Environ. Res. Public Health.* 17:6841.
33. Stepniak, J., A. Rynkowska, and M. Karbownik-Lewińska. 2022. Membrane lipids in the thyroid comparing to those in non-endocrine tissues are less sensitive to pro-oxidative effects of Fenton reaction substrates. *Front. Mol. Biosci.* 9, 901062.
34. Imai, T., S. Iwata, T. Hirayama, H. Nagasawa, S. Nakamura, M. Shimazawa…, 2019. Intracellular Fe2+ accumulation in endothelial cells and pericytes induces blood-brain barrier dysfunction in secondary brain injury after brain hemorrhage. *Sci. Rep.* 9:6228.
35. Mohanty, J. G., E. Nagababu, and J. M. Rifkind. 2014. Red blood cell oxidative stress impairs oxygen delivery and induces red blood cell aging. *Front. Physiol.* 5
36. Hebbel, R. P. 1991. Beyond hemoglobin polymerization: the red blood cell membrane and sickle disease pathophysiology. *Blood.* 77:214–237.
37. Stokes, G. Y., A. M. Buchbinder, J. M. Gibbs-Davis, K. A. Scheidt, and F. M. Geiger. 2008. Heterogeneous ozone oxidation reactions of 1-pentene, cyclopentene, cyclohexene, and a menthenol derivative studied by sum frequency generation. *J. Phys. Chem. A.* 112:11688–11698.
38. Voss, L. F., M. F. Bazerbashi, C. P. Beekman, C. M. Hadad, and H. C. Allen. 2007. Oxidation of oleic acid at air/liquid interfaces. *J. Geophys. Res. Atmos.* 112, D06209.
39. Tyrode, E., P. Niga, M. Johnson, and M. W. Rutland. 2010. Molecular structure upon compression and stability toward oxidation of Langmuir films of unsaturated fatty acids: a vibrational sum frequency spectroscopy study. *Langmuir.* 26:14024–14031.
40. Liljeblad, J. F. D., V. Bulone, E. Tyrode, M. W. Rutland, and C. M. Johnson. 2010. Phospholipid monolayers probed by vibrational sum frequency spectroscopy: instability of unsaturated phospholipids. *Biophys. J.* 98:L50–L52.
41. Qiao, L., A. Ge, M. Osawa, and S. Ye. 2013. Structure and stability studies of mixed monolayers of saturated and unsaturated phospholipids under low-level ozone. *Phys. Chem. Chem. Phys.* 15:17775–17785.
42. Qiao, L., A. Ge, Y. Lang, and S. Ye. 2015. Oxidative degradation of the monolayer of 1-Palmitoyl-2-Oleoyl-sn-Glycero-3-Phosphocholine (POPC) in low-level ozone. *J. Phys. Chem. B.* 119:14188–14199.
43. Inoue, K. I., C. Takada, L. Wang, A. Morita, and S. Ye. 2020. In situ monitoring of the unsaturated phospholipid monolayer oxidation in ambient air by HD-SFG spectroscopy. *J. Phys. Chem. B.* 124:5246–5250.
44. Inoue, K., K. Fujimoto, C. Takada, A. Ge, and S. Ye. 2021. Effect of head group on low-level ozone oxidation of unsaturated phospholipids on a water surface. *Bull. Chem. Soc. Jpn.* 94:486–489.
45. Qiao, L., Z. Chen, C. Takada, H. Chiba, K. Inoue, S.-P. Hui, and S. Ye. 2022. Quantitative evaluation on the degradation process of the pulmonary surfactant monolayer when exposed to low-level ozone of ambient environment. *Anal. Chem.* 94:8651–8658.
46. Wang, H. F., W. Gan, R. Lu, Y. Rao, and B. H. Wu. 2005. Quantitative spectral and orientational analysis in surface sum frequency generation vibrational spectroscopy (SFG-VS). *Int. Rev. Phys. Chem.* 24:191–256.
47. Wang, H.-F., L. Velarde, W. Gan, and L. Fu. 2015. Quantitative sum-frequency generation vibrational spectroscopy of molecular surfaces and interfaces: lineshape, polarization, and orientation. *Annu. Rev. Phys. Chem.* 66:189–216.
48. Hirose, C., N. Akamatsu, and K. Domen. 1992. Formulas for the analysis of surface sum-frequency generation spectrum by CH stretching modes of methyl and methylene groups. *J. Chem. Phys.* 96:997–1004.
49. Hirose, C., H. Yamamoto, N. Akamatsu, and K. Domen. 1993. Orientation analysis by simulation of vibrational sum frequency generation spectrum: CH stretching bands of the methyl group. *J. Phys. Chem.* 97:10064–10069.
50. Feng, R. J., X. Li, Z. Zhang, Z. Lu, and Y. Guo. 2016. Spectral assignment and orientational analysis in a vibrational sum frequency generation study of DPPC monolayers at the air/water interface. *J. Chem. Phys.* 145, 244707.
51. Fellows, A. P., M. T. L. Casford, and P. B. Davies. 2021. Orientation analysis of sum-frequency generation spectra of di-chain phospholipids: effect of the second acyl chain. *AIP Adv.* 11, 045119.
52. Fellows, A. P., M. T. L. Casford, and P. B. Davies. 2022. Investigating Bénard-Marangoni migration at the air-water interface in the time domain using sum frequency generation (SFG) of palmitic acid Monolayers. *J. Chem. Phys.* 156, 164701.
53. Dluhy, R. A. 2007. Infrared spectroscopy of biophysical monomolecular films at interfaces: theory and applications. *Appl. Spectrosc. Rev.* 35:315–351.
54. Siddique, Y. H., G. Ara, and M. Afzal. 2012. Estimation of lipid peroxidation induced by hydrogen peroxide in cultured human lymphocytes. *Dose-Reponse.* 10:1–10.
55. Sheridan, A. M., S. Fitzpatrick, C. Wang, D. C. Wheeler, and W. Lieberthal. 1996. Lipid peroxidation contributes to hydrogen peroxide induced cytotoxicity in renal epithelial cells. *Kidney Int.* 49:88–93.
56. Forman, H. J., A. Bernardo, and K. J. A. Davies. 2016. What is the concentration of hydrogen peroxide in blood and plasma? *Arch. Biochem. Biophys.* 603:48–53.
57. Kumar, S., D. K. Yadav, E.-H. Choi, and M.-H. Kim. 2018. Insight from molecular dynamic simulation of reactive oxygen species in oxidized skin membrane. *Sci. Rep.* 8, 13271.
58. Munro, S. 2003. Lipid rafts: elusive or illusive? *Cell.* 115:377–388.
59. Shoemaker, S. D., and T. K. Vanderlick. 2003. Material studies of lipid vesicles in the Lα and Lα-Gel coexistence regimes. *Biophys. J.* 84:998–1009.
60. Shimokawa, N., M. Nagata, and M. Takagi. 2015. Physical properties of the hybrid lipid POPC on micrometer-sized domains in mixed lipid membranes. *Phys. Chem. Chem. Phys.* 17:20882–20888.
61. Ziblat, R., L. Leiserowitz, and L. Addadi. 2010. Crystalline domain structure and cholesterol crystal nucleation in single hydrated DPPC:cholesterol:POPC bilayers. *J. Am. Chem. Soc.* 132:9920–9927.
62. Yusupov, M., K. Wende, S. Kupsch, E. C. Neyts, S. Reuter, and A. Bogaerts. 2017. Effect of head group and lipid tail oxidation in the cell membrane revealed through integrated simulations and experiments. *Sci. Rep.* 7:5761.
63. Kwan, W. P., and B. M. Voelker. 2002. Decomposition of hydrogen peroxide and organic compounds in the presence of dissolved iron and ferrihydrite. *Environ. Sci. & Technol.* 36:1467–1476.

64. Duesterberg, C. K., S. E. Mylon, and T. D. Waite. 2008. pH effects on iron-catalyzed oxidation using Fenton's reagent. *Environ. Sci. & Technol.* 42:8522–8527.
65. Vasquez-Medrano, R., D. Prato-Garcia, and M. Vedrenne. 2018. Advanced Oxidation Processes for Waste Water Treatment: Emerging Green Chemical Technology. Academic Press, Cambridge, MA, pp. 89–113.
66. Hamm, L. L., N. Nakhoul, and K. S. Hering-Smith. 2015. Acid-base homeostasis. *Clin. J. Am. Soc. Nephrol.* 10:2232–2242.
67. Zhang, Y., and M. Zhou. 2019. A critical review of the application of chelating agents to enable Fenton and fenton-like reactions at high pH values. *J. Hazard. Mater.* 362:436–450.
68. Klopfer, K. J., and T. K. Vanderlick. 1996. Isotherms of dipalmitoylphosphatidylcholine (DPPC) monolayers: features revealed and features obscured. *J. Colloid Interface Sci.* 182:220–229.
69. Venable, R. M., F. L. H. Brown, and R. W. Pastor. 2015. Mechanical properties of lipid bilayers from molecular dynamics simulation. *Chem. Phys. Lipids.* 192:60–74.
70. Shinoda, W. 2016. Permeability across lipid membranes. *Biochim. Biophys. Acta - Biomembr.* 1858:2254–2265.
71. Tim, B., P. Blaszkiewicz, and M. Kotkowiak. 2022. Altering model cell membranes by means of photoactivated organic functionalized gold nanorods. *J. Mol. Liq.* 349, 118179.
72. Wei, F., W. Xiong, W. Li, W. Lu, H. C. Allen, and W. Zheng. 2015. Assembly and relaxation behaviours of phosphatidylethanolamine monolayers investigated by polarization and frequency resolved SFG-VS. *Phys. Chem. Chem. Phys.* 17:25114–25122.
73. Liu, W., Z. Wang, L. Fu, R. M. Leblanc, and E. C. Y. Yan. 2013. Lipid compositions modulate fluidity and stability of bilayers: characterization by surface pressure and sum frequency generation spectroscopy. *Langmuir.* 29:15022–15031.
74. Niga, P., P. M. Hansson-Mille, A. Swerin, P. M. Claesson, J. Schoelkopf, P. A. C. Gane, E. Bergendal, A. Tummino, R. A. Campbell, and C. M. Johnson. 2018. Interactions between model cell membranes and the neuroactive drug propofol. *J. Colloid Interface Sci.* 526:230–243.
75. Levental, I., P. A. Janmey, and A. Cebers. 2008. Electrostatic contribution to the surface pressure of charged monolayers containing polyphosphoinositides. *Biophys. J.* 95:1199–1205.
76. Bañuelos-Frias, A., V. M. Castañeda-Montiel, E. R. Alvizo-Paez, E. A. Vazquez-Martinez, E. Gomez, and J. Ruiz-Garcia. 2021. Thermodynamic and mechanical properties of DMPC/cholesterol mixed monolayers at physiological conditions. *Front. Phys.* 9, 636149.
77. Volinsky, R., R. Paananen, and P. K. J. Kinnunen. 2012. Oxidized phosphatidylcholines promote phase separation of cholesterol-sphingomyelin domains. *Biophys. J.* 103:247–254.
78. Pogodin, S., and V. Baulin. 2010. Coarse-grained models of biological membranes within the single chain mean field theory. *Soft Matter.* 6:2216–2226.
79. Choudhary, K., J. Kumar, P. Taneja, R. K. Gupta, and V. Manjuladevi. 2017. Langmuir–Blodgett films of stearic acid deposited on substrates at different orientations relative to compression direction: alignment layer for nematic liquid crystal. *Liq. Cryst.* 44:1592–1599.
80. Akamatsu, N., K. Domen, and C. Hirose. 1993. SFG study of two-dimensional orientation of surface methyl groups on cadmium arachidate Langmuir-BIodgett films. *J. Phys. Chem.* 97:10070–10075.
81. Ma, G., and H. C. Allen. 2006. DPPC Langmuir monolayer at the air-water interface: Probing the tail and head groups by vibrational sum frequency generation spectroscopy. *Langmuir.* 22:5341–5349.
82. Cernescu, A., M. Szuwarzyński, U. Kwolek, P. Wydro, M. Kepczynski, S. Zapotoczny, M. Nowakowska, and L. Quaroni. 2018. Label-free infrared spectroscopy and imaging of single phospholipid bilayers with nanoscale resolution. *Anal. Chem.* 90:10179–10186.
83. Derenne, A., T. Claessens, C. Conus, and E. Goormaghtigh. 2013. Encyclopedia of Biophysics. Springer, Berlin, pp. 1074–1081.
84. Oleszko, A., S. Olsztyńska-Janus, T. Walski, K. Grzeszczuk-Kuć, J. Bujok, K. Gałecka, A. Czerski, W. Witkiewicz, and M. Komorowska. 2015. Application of FTIR-ATR spectroscopy to determine the extent of lipid peroxidation in plasma during haemodialysis. *Biomed Res. Int.* 2015:1–8.
85. Gaber, B. P., P. Yager, and W. L. Peticolas. 1978. Conformational nonequivalence of chains 1 and 2 of dipalmitoyl phosphatidylcholine as observed by Raman spectroscopy. *Biophys. J.* 24:677–688.
86. Abramson, M. B., W. T. Norton, and R. Katzman. 1964. Study of ionic structures in phospholipids by infrared spectra. *J. Biol. Chem.* 240:2389–2395.
87. Schultz, Z. D., and I. W. Levin. 2011. Vibrational spectroscopy of biomembranes. *Annu. Rev. Anal. Chem.* 4:343–366.
88. Zeiss, H. H., and M. Tsutsui. 1953. The carbon-oxygen absorption band in the infrared spectra of alcohols. *J. Am. Chem. Soc.*
89. Smith, B. 1998. Infrared Spectral Interpretation: A Systematic Approach. CRC Press, Boca Raton, pp. 67–90.
90. Coates, J. 2000. Encyclopedia of Analytical Chemistry. Wiley, Hoboken, pp. 10815–10837.
91. Gericke, A., and R. Mendelsohn. 1996. Partial chain deuteration as an IRRAS probe of conformational order of different regions in hexadecanoic acid monolayers at the air/water interface. *Langmuir.* 12:758–762.
92. Collin, F. 2019. Chemical basis of reactive oxygen species reactivity and involvement in neurodegenerative diseases. *Int. J. Mol. Sci.* 20:2407.
93. Juan, C. A., J. M. P. delaLastra, F. J. Plou, and E. Pérez-Lebeña. 2021. The chemistry of reactive oxygen species (ROS) revisited: outlining their role in biological macromolecules (DNA, lipids and proteins) and induced pathologies. *Int. J. Mol. Sci.* 22:4642.

Biophysical Journal, Volume *122*

Supplemental information

In situ investigation of the oxidation of a phospholipid monolayer by reactive oxygen species

Alexander P. Fellows, Mike T.L. Casford, and Paul B. Davies

Supplemental Information: *In situ* Investigation of the Oxidation of a Phospholipid Monolayer by Reactive Oxygen Species (ROS)

Alexander P. Fellows*,1 Mike T. L. Casford,1 and Paul B. Davies,1

1: Yusuf Hamied Department of Chemistry, University of Cambridge, Cambridge, CB21EW, United Kingdom

* apf36@cam.ac.uk

SFG Fitting Parameters

As mentioned in materials and methods, the SFG spectra were fitted according to the SFG equation with the terminal methyl groups having C_s symmetry. SFG simulations using this symmetry have shown that the in-plane mode contributes no intensity to the SSP polarisation combination and is negligible in PPP compared to the contributions from the out-of-plane mode.(1, 2) Therefore, as these are the only two polarisation combination employed in this work, for the purposes of fitting, only a single antisymmetric methyl resonance (r^-_{OP}) was fitted.

SFG spectra were fitted using an in-house MATLAB script using the parameters given in Table S1. The monolayers were assumed to adopt an all-trans geometry with 0° twist angles and have orientational distributions described by delta functions. By convention these assumptions are considered to be acceptable for well-packed monolayers and are commonplace in SFG investigations on lipids in the liquid-condensed phase.

Table S1: Parameters used for SFG fitting.

Parameter	Description	Value
n_1^{IR}	Refractive Index of IR in Air-Phase	1.00
n_1^{VIS}	Refractive Index of VIS in Air-Phase	1.00
n_1^{SFG}	Refractive Index of SFG in Air-Phase	1.00
n_2^{IR}	Refractive Index of IR in Sub-Phase	1.390 + 0.013i
n_2^{VIS}	Refractive Index of VIS in Sub-Phase	$1.326 + 1.3\times10^{-7}i$
n_2^{SFG}	Refractive Index of SFG in Sub-Phase	1.331

n_{layer}	Refractive Index of Monolayer	1.18
θ_{IR}	Incident IR Beam Angle	55°
θ_{VIS}	Incident VIS Beam Angle	60°
θ_{SFG}	Output SFG Beam Angle	59.27-59.31°
λ_{VIS}	VIS Beam Wavelength	532 nm
r	Single Bond Polarisability Derivative Ratio	0.0

SFG Analysis of the Phosphate Stretching Region

FTIR spectra shown in the manuscript led to the conclusion that the dominant chemical changes from ROS attack occur to the lipid head-group, in particular indicating changes to the phosphate and choline functional groups. It should, however, be noted that SFG spectra in both the SSP and PPP polarisation combinations showed no resonances from 1050-1600 cm^{-1}. (SFG spectra of phospholipids in this lower frequency range have been recorded previously(3) where bands such as the phosphate stretching modes have been observed.) The SFG spectrometer employed here, however, has a reduced output IR power at these lower IR frequencies (by ~1 order of magnitude compared to the C-H stretching region). Therefore, when combined with the relatively low acquisition rate (50 Hz), the usually weak phosphate bands become indistinguishable from the baseline.

Kinetic Mechanism of Fenton's Reagent

Catalytic activity in Fenton's reagent is often attributed to Fe^{2+} ions which can be regenerated from Fe^{3+} ions in steps 1 and 2 shown in the main text. The data in Figure 4 shows that the monolayer is sensitive to the sub-phase pH which causes precipitation of ferrous or ferric hydroxides. Furthermore, an increased concentration of $FeSO_4$ in the sub-phase (100 mg dm^{-3} cf. 10 mg dm^{-3}) showed a greater reduction in surface pressure from the same amount of injected H_2O_2 (e.g., by comparing Figures 3 and 5), pointing to non-catalytic behaviour.

Impact of Fe^{2+} Concentration on the Isotherm Changes

The effect of [$FeSO_4$] on the extent of lipid oxidation for equivalent amounts of injected H_2O_2 is shown in Figure S1 which plots isotherms recorded every 20 minutes after the injection of

H_2O_2 as presented in Figure 5 but for four further sub-phase $FeSO_4$ concentrations, ranging from 20-300 mg dm^{-3}.

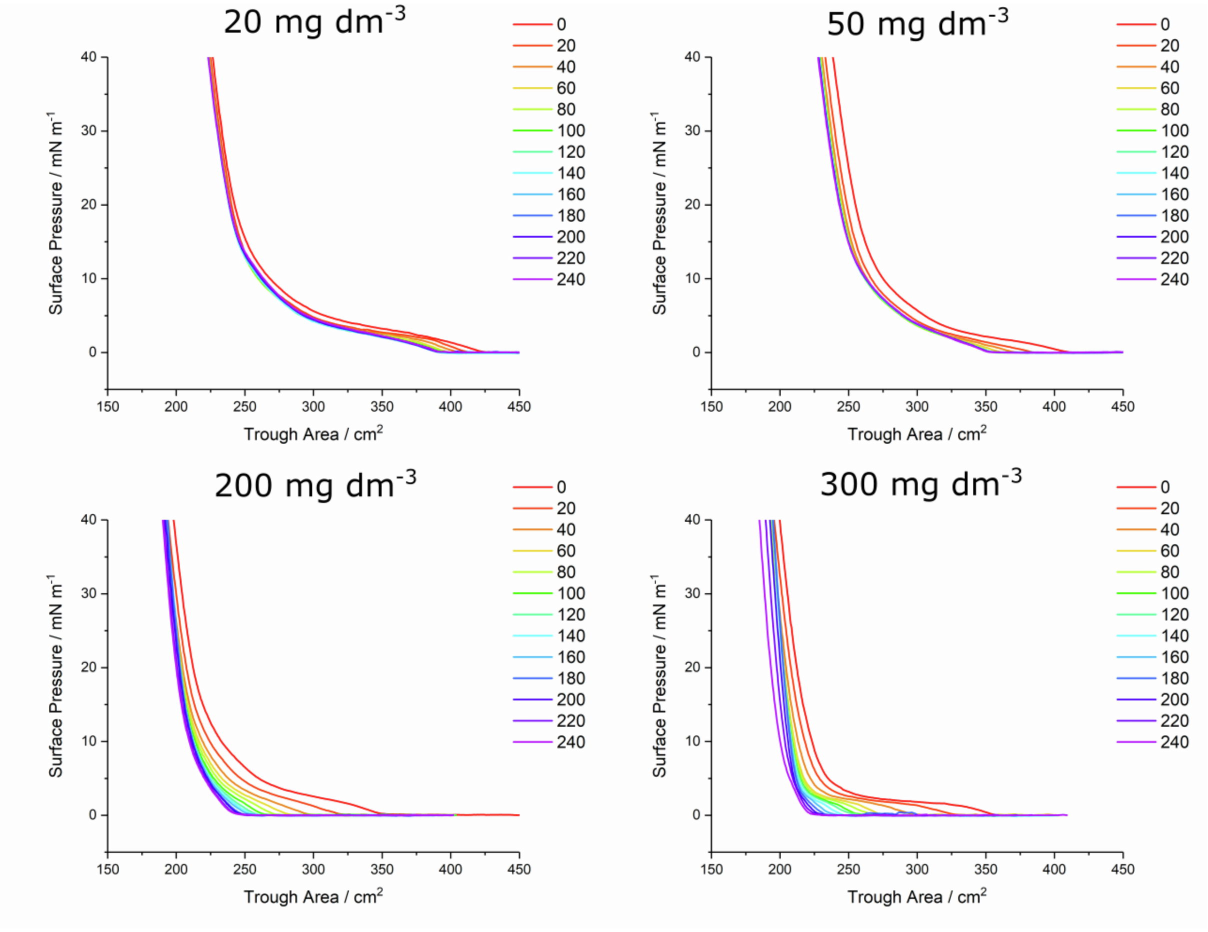

Figure S1: (Π – A) isotherms of a DPPC monolayer recorded at 20-minute intervals after the injection of H_2O_2 into the sub-phase containing different concentrations of $FeSO_4$, from 20 – 300 mg dm^{-3}.

Figure S2 shows the temporal change in trough area at 1 mN m^{-1} surface pressure (Figure S2a) and film compressibility (Figure S2b) for different $FeSO_4$ concentrations, the latter arising from the gradient between 30 and 40 mN m^{-1} from the data in Figure S1. Also shown are the equilibrium values reached for both parameters (Figure S2c and d), calculated from fits to an exponential decay of the plots in Figure S2a and taking the values reached after 4 hours in Figure S2b. The monolayer seems to become more condensed (occupying less surface area) and less compressible (showing a steeper gradient on compression). It is also worth noting that the change in compressibility is not monotonic, but the gradient appears to sharpen (increase) and then become shallower (decrease), as seen in Figure S2b, particularly at higher concentrations of $FeSO_4$. This correlates well with the multi-step kinetic mechanism

proposed from the temporal SFG and IRRAS analysis in the main text. Nevertheless, the differing concentrations of $FeSO_4$ seem to result in equivalent monolayer changes, only to different extents.

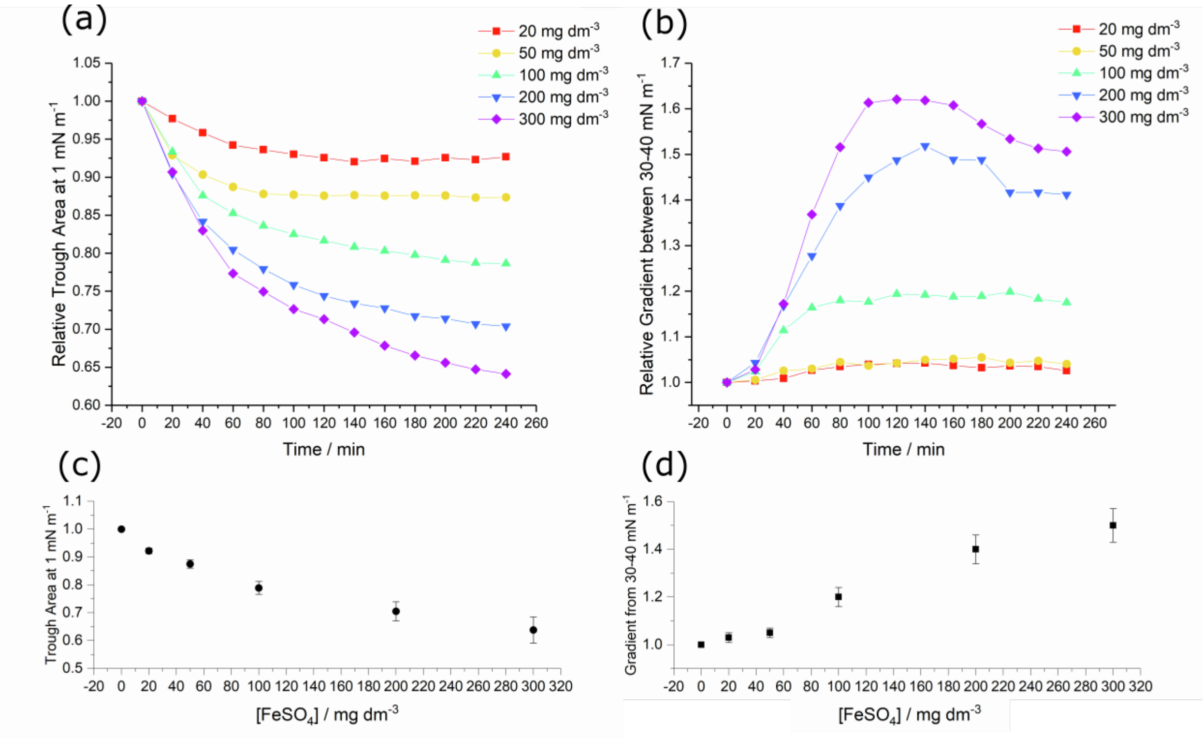

Figure S2: Analysis of the $\Pi - A$ isotherms shown in Figure S1 for different concentrations of $FeSO_4$ in the sub-phase showing: (a) the change in relative trough area at 1 mN m^{-1}, (b) the relative change in isotherm gradient between 30-40 mN m^{-1}, (c) the equilibrium values of trough area at 1 mN m^{-1} calculated by fitting the plots in (a) to exponential decays, and (d) the final values of the gradient between 30 and 40 mN m^{-1}, taken as the values 4 hours after the injection of H_2O_2.

A Kinetic Description of Fenton Lipid Oxidation

Figure S2 shows that greater concentrations of aqueous ferrous ions in the sub-phase yield more substantial changes in the monolayer. Given that the ferrous salts are considered catalytic, it might be expected that a change in their concentration would result in a more rapid oxidation of the monolayer (i.e., higher decay constants), but the final changes should remain the same as they are dependent only on the amount of H_2O_2 available for conversion into ROS radicals. Evidently, this is not what is happening. Not only are the changes more significant for the same amount of H_2O_2 added, but the decay constants are smaller for higher $FeSO_4$ concentrations and take longer to reach their final values (Figure S2a). These observations could point to the

ferrous salts not acting solely as catalysts but are also being consumed in the reaction. This could be due to a combination of poor water solubility of ferric salts and hence precipitating out as ferric hydroxide, and not regenerating Fe^{2+} (i.e. $k_2 \rightarrow 0$), or a chain termination step as suggested in the Haber-Weiss reaction:

$$Fe^{2+} + OH + H^{+} \xrightarrow{k_3} Fe^{3+} + H_2O$$

where ferrous ions are converted to ferric ions and where OH radicals are destroyed rather than created, which would correspond to an inhibition step.

The precipitation of ferric hydroxide is supported by the observed colour change from nearly colourless to orange after the injection of H_2O_2. This implies that the conversion of Fe^{2+} to Fe^{3+} is irreversible. The decrease in observed decay constants at higher $FeSO_4$ concentrations (Figure S2a) indicates the significance of the inhibition step. By considering an elementary kinetic mechanism, based on the two steps given in the manuscript (with rate constants k_1 and k_2) and third step presented above (with rate constant k_3), as well as the direct lipid oxidation step:

$$L + OH \xrightarrow{k_L} L_{ox}$$

it is straightforward to show that the rate of loss of lipid is given by Eq. S1. In the absence of the inhibition step (i.e., $k_3 = 0$), the rate is simply determined by step 1 and is dependent only on the concentrations of Fe^{2+} and H_2O_2. However, including the inhibition step reduces the reaction rate, where it is clear that higher Fe^{2+} concentrations inhibit the oxidation of lipids (as $[Fe^{2+}]$ is included in the k_3 term in the denominator). Under conditions where ferric hydroxide is precipitated, the reaction becomes dependent on the *initial* concentration of $FeSO_4$ since ferrous ions cannot be regenerated.

$$\frac{d[L]}{dt} = -\frac{k_1 k_L [L][Fe^{2+}][H_2O_2]}{k_3[Fe^{2+}][H^{+}] + k_L[L]} \qquad (S1)$$

To further demonstrate how the extent of oxidation depends on both the $FeSO_4$ and H_2O_2 concentrations, the resulting pressure drop of a DPPC monolayer starting at 20 mN m^{-1} was monitored whilst varying the concentrations of each reactant in the sub-phase (i.e., both

the starting $FeSO_4$ concentration and the amount of H_2O_2 added). The resulting behaviour of the film is shown in Figure S3. On increasing the initial $FeSO_4$ concentration, the increase in the pressure drop becomes smaller, in agreement with the above analysis which suggested that Fe^{2+} cannot be regenerated and therefore inhibits lipid oxidation. Significantly, Figure S3b shows there is a levelling off of saturation in the pressure drop at high H_2O_2 concentrations, suggesting that H_2O_2 is in excess when added in these amounts and that the Fe^{2+} in the reaction mixture has been consumed.

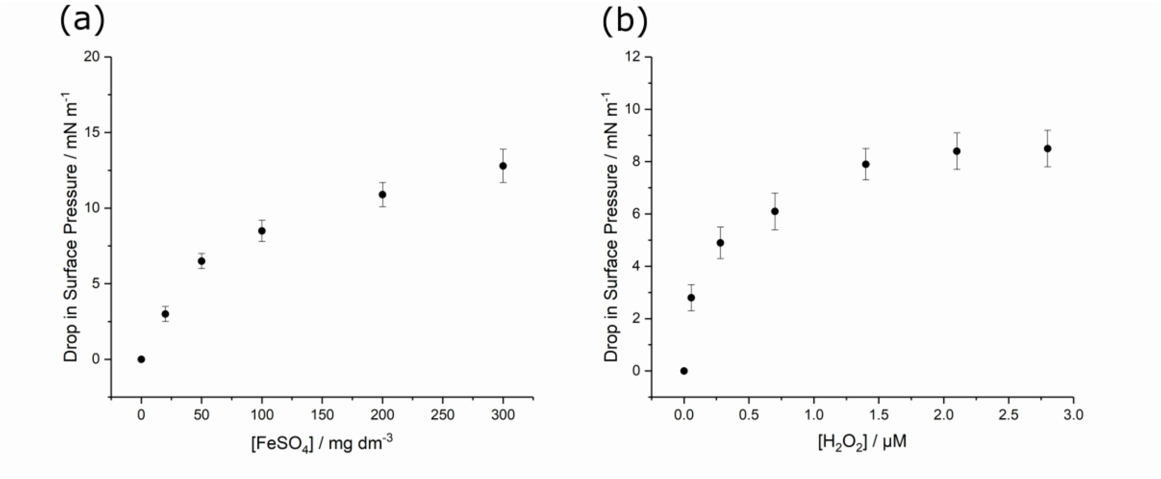

Figure S3: Drop in surface pressure for a 20 mN m^{-1} DPPC monolayer at the air- $FeSO_{4(aq)}$ interface in response to the injection of H_2O_2 into the sub-phase showing: (a) the effect of changing the sub-phase $FeSO_4$ concentration, and (b) the effect of changing the amount of H_2O_2 injected (converted into the equivalent concentration in the sub-phase).

References

1. Feng, R.J., X. Li, Z. Zhang, Z. Lu, and Y. Guo. 2016. Spectral assignment and orientational analysis in a vibrational sum frequency generation study of DPPC monolayers at the air/water interface. *J. Chem. Phys.* 145:244707.

2. Fellows, A.P., M.T.L. Casford, and P.B. Davies. 2021. Orientation Analysis of Sum-Frequency Generation Spectra of Di-chain Phospholipids: Effect of the Second Acyl Chain. *AIP Adv.* 11:045119.

3. Ma, G., and H.C. Allen. 2006. DPPC Langmuir monolayer at the air-water interface: Probing the tail and head groups by vibrational sum frequency generation spectroscopy. *Langmuir*. 22:5341–5349.
